# *In Vitro* models of leukemia development: the role of very small leukemic stem-like cells in the cellular transformation cascade

**DOI:** 10.3389/fcell.2024.1463807

**Published:** 2025-01-03

**Authors:** Jan Jakub Lica, Joanna Jakóbkiewicz-Banecka, Andrzej Hellmann

**Affiliations:** ^1^ Department Medical Biology and Genetics, Faculty of Biology, University of Gdansk, Gdansk, Poland; ^2^ Department Health Science; Powiśle University, Gdańsk, Poland; ^3^ Department of Hematology and Transplantology, Faculty of Medicine, Medical University of Gdansk, Gdańsk, Poland

**Keywords:** very small progenitor and precursor stem cells, very small leukemic stem-like cells, acute myeloid leukemia development *in vitro*, cellular transformations, HL60, A549

## Abstract

Recent experimental findings indicate that cancer stem cells originate from transformed very small embryonic-like stem cells. This finding represents an essential advancement in uncovering the processes that drive the onset and progression of cancer. In continuously growing cell lines, for the first time, our team’s follow-up research on leukemia, lung cancer, and healthy embryonic kidney cells revealed stages that resembles very small precursor stem cells. This review explores the origin of leukemic stem-like cells from very small leukemic stem-like cells establish from transformed very small embryonic-like stem cells. We explore theoretical model of acute myeloid leukemia initiation and progresses through various stages, as well basing the HL60 cell line, present its hierarchical stage development *in vitro*, highlighting the role of these very small precursor primitive stages. We also discuss the potential implications of further research into these unique cellular stages for advancing leukemia and cancer treatment and prevention.

## Background

The earliest experimental evidence supporting the presence of malignancy stem cells (MSCs) in humans was reported in 1994 (Lapidot et al., 1994) and has since been the subject of extensive research, contributing to a better understanding of tumorigenesis and the development of more effective anti-malignancy therapies (Clarke and Fuller, 2006; Fulawka et al., 2014; Yadav and Desai, 2019; Lica et al., 2024; Zhang et al., 2024). Stem cells (SCs) are undifferentiated, primitive cells in an organism (Fuchs and Segre, 2000; Weissman, 2000; Bongso and Lee, 2005; Brignier and Gewirtz, 2010). When cultured *in vitro*, these cells often undergo epigenetic changes and exhibit dysfunctions; therefore, they are better described as stem-like cells (SLCs) (Nestor et al., 2015; Weissbein et al., 2017; Bar and Benvenisty, 2019; Liu et al., 2020; Tognon et al., 2021; Kabacik et al., 2022).

The initial evidence suggesting the existence of SCs in the human body can be traced back to a study published in 1959, which demonstrated that transplanted bone marrow cells could regenerate the hematopoietic system in patients who had undergone intensive whole-body irradiation, indicating the existence of hematopoietic SCs (HSCs) (Thomas et al., 1959). The first detailed description of healthy HSCs derived from long-term *in vitro* cultures was published in 1980, where they were referred to as long-term culture-initiating cells (Gartner and Kaplan, 1980). Today, they would more accurately be called hematopoietic SLCs (HSLCs).

In cancer research, a subset of SCs has also been identified. Malignant cells (MCs) originating from epithelial tissues are known as cancer SCs (CSCs), while those from other germ layers have specific names; for example, in leukemia, they are termed leukemic SCs (LSCs) (Lapidot et al., 1994). The CSC hypothesis was initially considered controversial (Bhagwandin and Shay, 2009; Magee et al., 2012) but has gained broad acceptance over time (Batlle and Clevers, 2017; Phi et al., 2018; Najafi et al., 2019; Saygin et al., 2019; Liu et al., 2023). Outside the organism, MSCs, including CSCs and LSCs, are called cancer SLCs (CSLCs) (Kondo et al., 2004) or leukemic SLCs (LSLCs) (Bonnet and Dick, 1997).

Evidence for the presence of LSLCs in cell lines emerged in the 1990s and 2000s, primarily in studies focused on leukemia cell lines such as HL60 (Bonnet and Dick, 1997). The stage-specific heterogeneity of this and other acute myeloid leukemia (AML) cell lines enabled the identification of a small subpopulation of LSLCs. These cells exhibited symmetric division (SD) capabilities, the ability to initiate and drive disease progression, and cytological and morphological similarities to LSCs (Lapidot et al., 1994; Bonnet and Dick, 1997; Guan et al., 2003). Notably, the HL60 cell line was established in the 1970s through the leukapheresis of peripheral blood from a patient with AML, and even at that time, the presence of cells with LSLC properties capable of initiating AML in NOD mice was suggested (Collins et al., 1977; Gallagher et al., 1979). The first HL-60 leukopoiesis model was proposed in 1988, identifying LSLCs at the top of the hierarchical development of this cell line (Birnie, 1988), which appears to support subsequent mathematical models of hematopoietic senescence (Marciniak-Czochra et al., 2009; Stiehl et al., 2014).

Following the discovery of LSLCs, CSLCs were identified in cultures from solid tumors, including those from brain and breast cancers (Ignatova et al., 2002; Al-Hajj et al., 2003; Singh et al., 2004). Glioblastoma CSCs (U87) were discovered in 2002, and they had been characterized using the *PROM1* marker (formerly known as cluster of differentiation 133 - CD133) by 2004 (Ignatova et al., 2002; Singh et al., 2004). These cells demonstrated the capacity to SD, differentiate into various cell types, and initiate tumor growth *in vivo* when transplanted into animals, exhibiting traits similar to neural SCs. In 2003, CSLCs were isolated from the MCF7 breast cancer cell line and identified by the surface markers CD44⁺/CD24⁻ (Al-Hajj et al., 2003). These cells exhibited a tumor-initiating capacity in immunodeficient mice and shared key CSC features, such as SD and the ability to differentiate into multiple lineages.

Unlike multipotent SCs such as HSCs, pluripotent SCs (PLSCs) can develop into all cell types in the body, distinguishing them from tissue-specific SCs such as HSCs. Human PLSCs from an embryo, called embryonic stem cells (ESCs), were first successfully isolated and described in 1998 (Thomson et al., 1998). Shortly thereafter, *in vitro* cultures containing equivalent cells, termed pluripotent-like SCs and embryonic-like SCs (ELSCs), were established (Amit et al., 2000). In 2007, induced PLSCs (iPLSCs) were created from adult human fibroblasts through reprogramming with key transcription factors (octamer-binding transcription factor 4 [OCT4], SRY-box transcription factor 2 [SOX2], Kruppel-like factor 4 [KLF4], and MYC protooncogene [c-MYC]). These iPLSCs behave like ESCs and can differentiate into various cell types (Takahashi et al., 2007).

One of the most primitive ESCs in the human body are cells derived from the inner cell mass (ICM) of the blastocyst. Epiblast cells (EpibCs) form from the ICM in the pre-gastrulation stage and play a key role in early embryo development (Tam and Loebel, 2007; Rossant, 2008). EpibCs are the source of the germ layers (ectoderm, mesoderm, and endoderm), as well as extraembryonic cells, which forms structures supporting embryo development, including the chorion (a part of the placenta) and the allantois (Arnold and Robertson, 2009; Hayashi and Saitou, 2013; Saitou and Miyauchi, 2016).

The EpibCs also generate primordial germ cells (PGCs), which in humans appear around the second to third week of embryonic development, originating from the epiblast. PGCs migrate from the posterior part of the embryo to the developing gonads (testes or ovaries), where they later differentiate into gametes. Studies on human embryos have shown that PGCs emerge early in development and are crucial for forming the germline. These cells play vital roles in embryo development and the formation of supporting structures, enabling proper fetal development (Rossant and Tam, 2017).

**FIGURE 1:**
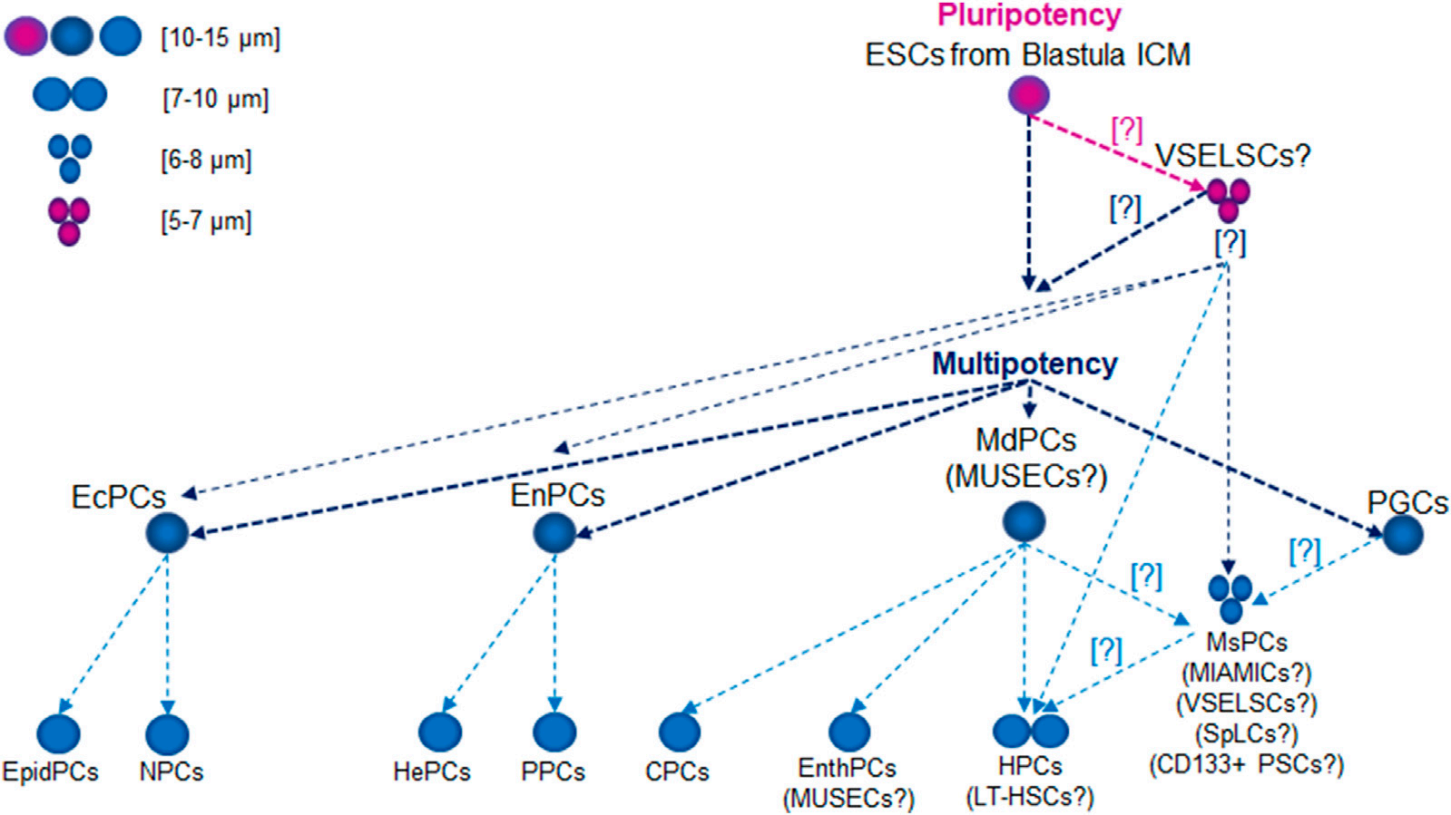
Lifetime Development of Primitive Stem Cell Precursors and Progenitors. Abbreviations: CPCs, cardiac precursor cells; CD133+ PSCs, CD133+ precursor cells; EcPCs, ectodermal progenitor cells; EnthPC, endothelial progenitor cells; EnPCs, endodermal progenitor cells; EpidPCs, epidermal precursor cells; ESCs, embryonic stem cells; HePCs, hepatic precursor cells; HPC, hematopoietic precursor cells; ICM, inner cell mass; LT-HSCs, long-term hematopoietic stem cells; MdPCs, mesoderm progenitor cells; MIAMICs, marrow-isolated adult multilineage inducible cells; MsPCs, endothelial progenitor cells; MUSECs, multilineage-differentiating stress enduring cells; NPCs, neural precursor cells; PGCs, primordial germ cells; PPCs, pancreatic progenitor cells; SpLCs, spore-like cells; VSELSCs, very small embryonic-like stem cells.

During gastrulation, EpibCs migrate into the embryo, giving rise to ectodermal progenitor cells (EcPCs), mesoderm progenitor cells (MdPCs), endodermal progenitor cells (EnPCs), and PGCs (Tam and Behringer, 1997). EcPCs give rise to neural precursor cells, which differentiate into various nervous system cells, including neurons, astrocytes, and oligodendrocytes, as well as epidermal precursor cells, which form skin, epidermal, and glandular cells. EnPCs are responsible for forming the digestive and respiratory systems, including the liver, pancreas, and lungs, through pathways such as hepatic and pancreatic progenitor cells. MdPCs develop into progenitor and precursor cells: hematopoietic precursor cells (HPCs), which will give rise to blood and lymphatic cells; mesenchymal precursor cells (MsPCs), responsible for forming muscle, bone, cartilage, and adipose tissue; cardiac precursor cells, which generate various heart cells; and endothelial progenitor cells (EnthPCs), involved in blood vessel formation (Figure 1).

Embryonic cancer cells (ECCs), also called embryonal carcinoma cells, are cancer cells that originate from embryonic tumors, particularly those related to germ cell tumors (germinomas) or other cancers developing from PGCs, such as embryonal carcinoma. ECCs were first mentioned in research on germ cell tumors, particularly mouse embryonal carcinoma (Stevens, 1958). These cells were identified and described in teratocarcinoma: tumors derived from pluripotent cells that can differentiate into various tissue types. Further studies on teratocarcinoma revealed that ECCs represent the malignant counterpart of pluripotent cells, maintaining the ability to SD and differentiate like normal ECs, but their growth is uncontrolled (Pierce et al., 1959). Early research on ECCs focused on tumors linked to the gonads (Damjanov and Solter, 1974). These ECCs arise from PGCs, which typically develop into gametes like sperm or eggs. When these cells undergo abnormal development or mutations, they can cause cancer, forming germ cell tumors, including embryonal carcinomas (Moraru et al., 2023). These tumors are mostly found in the testes (testicular cancer) or ovaries but can also occur in other regions, such as the mediastinum or brain, as extragonadal germ cell tumors like teratomas (Miller et al., 2014; Zhu et al., 2018; Kidder, 2024). ECCs are pluripotent, meaning they can differentiate into many cell types, similar to normal SCs (Cunningham et al., 2012; Gropp et al., 2012; Montilla-Rojo et al., 2023). However, unlike healthy SCs, ECCs grow uncontrollably, leading to cancer development. ECCs contribute significantly to the composition of heterogeneous tumors, such as teratomas and mixed embryonal carcinomas, which contain various tissues.

The precursor stages of MSCs have garnered significant interest, particularly in exploring the mechanisms of carcinogenesis (Bhartiya et al., 2023b). Recent experimental evidence indicates that CSCs can originate from malignantly transformed very small ELSCs (VSELSCs) (Bhartiya et al., 2023b).

The existence of CSCs from transformed VSELSCs *in vivo* has been exemplified by very small CSLCs (VSCSLCs) and LSLCs (VSLSLCs) observed *in vitro* (Lica and Pradhan, 2023). Notably, VSELSCs were discovered in the human body in 2007 (Kucia et al., 2007) and, like CSCs and SCs, were initially met with skepticism. However, numerous independent laboratories have since confirmed their existence *in vivo* (Ratajczak et al., 2019), and recent reports about their cell culture counterparts have generated considerable interest (Lica and Pradhan, 2023). The latter has opened up avenues for investigating leukemic and cancer SCs precursors *in vitro*, given the availability of experimental material in the form of cell cultures and the potential for efficient methods to enrich the number of primitive stages (Lica et al., 2018, 2021; Lica and Pradhan, 2023). There is a promising opportunity to compare the most primitive stages of healthy cells and MCs by obtaining a minimum of 5,000 (preferably 10,000) cell stages for single-cell RNA sequencing (scRNA-seq) analysis (Danielski, 2023). These results increase our chances of understanding the molecular mechanisms underlying malignant proliferation, as well as differences in cell cycle checkpoints (Skladanowski et al., 2009) and the types of cell stage division according to the Hayflick effect (HE) (Hayflick, 1965) between healthy VSELSCs and transformed VSCSCs as well as VSLSCs, along with their stage *in vitro* analogs. The abbreviations currently used and suggested for very small SC progenitors and precursors *in vivo* and *in vitro* are presented in Table 1.

**TABLE 1:** Very primitive cellular stages *in vitro* and *in vivo*.

Human cellular stage	
*In vivo* (year of discovery)	*In vitro* (year of discovery)
ESCs: embryonal stem cells (1998)	ELSCs: embryonal-like stem cells (2000)
HSCs: hematopoietic stem cells (1959)	HSLCs: hematopoietic stem-like cells (1980)
CSCs: cancers stem cells (2002)	CSLCs: cancers stem-like cells (2003)
LSCs: leukemic stem cells (1994)	LSLCs: leukemic stem-like cells (1997)
VSELSCs/VSELs: very small embryonic-like stem cells (2006)	VSESLCs: very small embryonic stem-like cells (2023)
VSCSCs: very small cancer stem cells (2023)^a^	VSCSLCs: very small cancer stem-like cells (2023) VSLSLCs: very small leukemic stem-like cells (2023)

ESCs: (Thomson et al., 1998); ELSCs: (Amit et al., 2000); HSCs: (Thomas et al., 1959); HSLCs: (Gartner and Kaplan, 1980); CSCs: (Ignatova et al., 2002); CSLCs: (Al-Hajj et al., 2003); LSCs: (Lapidot et al., 1994); LSLCs: (Bonnet and Dick, 1997); VSELSCs: (Kucia et al., 2006); VSLSLCs: (Lica and Pradhan, 2023); VSCSLCs: (Lica and Pradhan, 2023); VSLSLCs: (Lica and Pradhan, 2023).

^a^ VSCSCs: Indicating that CSCs originate from VSELSCs (Bhartiya et al., 2023b).

## Origin of VSELSCs

Independent studies have detected biological material morphologically typical of VSELSCs in human tissues across various age groups, from young to elderly individuals (Virant-Klun et al., 2008; Sovalat et al., 2011, 2016; Chang et al., 2014; Vojnits et al., 2014; Kakavoulia, 2021). In suitable experimental models, VSELSCs have exhibited pluripotent or multipotent capabilities (Bhartiya et al., 2012; Vojnits et al., 2014; Bhartiya, 2015), differentiating into various cell types, including skeletal cells (Havens et al., 2012), vascular endothelial cells, cardiomyocytes (Wu et al., 2011), lung epithelial cells (Jin et al., 2013), and male or female gametes (Virant-Klun et al., 2008, 2013; Bhartiya et al., 2012; Bhartiya, 2015).

However, critics argue that VSELSCs lack pluripotent characteristics (Danova-Alt et al., 2012; Ivanovic, 2012; Alvarez-Gonzalez et al., 2013; Szade et al., 2013). Supporters of VSELSCs contend that discrepant findings may arise from differences in research protocols.

They also emphasize that VSELSCs share molecular similarities with early-stage migratory PGCs, which, upon restoration of somatic genomic imprinting, resemble EpibCs capable of proliferating *in vitro* and differentiating into all three germ layers (Shin et al., 2010). It is hypothesized that VSELSCs may originate from PGCs, derived from the EpibCs during the early stages of embryogenesis, typically within the first weeks of human development (Kucia M. et al., 2008; Ratajczak et al., 2013b; Ratajczak et al., 2019; Barati et al., 2021; Bhartiya et al., 2022; Bhartiya et al., 2023a). PGCs migrate to the developing gonads, where they differentiate into spermatogonia (sperm precursors) in males or oogonia (egg precursors) in females. When isolated and cultured under specific conditions, primordial germ-like cells (PGLCs) can be reprogrammed into pluripotent embryonic germ-like cells (EGLCs), which can differentiate into various cell types, like ESCs, albeit with epigenetic differences (Durcova-Hills et al., 2008; Panula et al., 2010; Nagamatsu et al., 2013; Kurek et al., 2020; Tran et al., 2021; Jo et al., 2022; Reda et al., 2022). The size of human ESLCs and PGLCs depends on their developmental stage and culture conditions. ESLCs, with an average size of 10–15 μm, are small with large nuclei due to their high metabolic activity and rapid division. PGLCs, slightly larger at 12–20 μm, exhibit size variations influenced by culture conditions and developmental cues, reflecting their preparation for migration and differentiation observed in early embryogenesis.

Recent research has demonstrated methods for differentiating human SCs into PGLCs, which mimic the behavior of PGCs during embryogenesis, particularly when cultured with specific signals such as bone morphogenetic protein 4 (BMP4) and Nodal protein (NODAL) (Shamblott et al., 1998). This differentiation allows the early steps of human germ cell development to be studied even though these cells do not exist naturally in adult human bodies. PGLCs have a limited capacity for directly forming tissue-specific SLCs; however, their pluripotent descendants, such as EGLCs, can differentiate into various tissue types *in vitro* (Yu et al., 2007; Durcova-Hills et al., 2008; Panula et al., 2010; Nichols and Smith, 2012; Nagamatsu et al., 2013; Katajisto et al., 2015). The observed pluripotency of cells exhibiting the cytological features of VSELSCs indicates that their proliferative potential exceeds that of PGCs.

The asymmetric division (AD) of PGCs has been well-studied in mice, although research on humans is more limited (Rossant and Tam, 2009; Bedzhov and Zernicka-Goetz, 2014). Much of what is known about PGC mechanisms comes from indirect observations and *in vitro* studies of PLGCs. Studies have shown that these cells undergo ADs, leading to gamete differentiation (Surani et al., 2007; Perrett et al., 2008; Gkountela et al., 2013; Tang et al., 2016). In humans, this suggests that PLGC division results in one retained SC and another differentiating cell (Tang et al., 2016). The retained SC continues to divide and differentiate into germ cells, while the daughter cell begins differentiating into more mature forms of germ cells, such as oogonia in females or spermatogonia in males. The ADs of PGCs and PLGCs ensure the SC pool is maintained while allowing other cells to differentiate into more advanced developmental stages (Gkountela et al., 2013; Tang et al., 2016). Research on SC dynamics has explored how the balance between SD and AD can fluctuate based on environmental cues and developmental stages. SD may be activated to replenish lost SCs during injury or periods of rapid growth, while maintaining homeostasis often requires AD to prevent uncontrolled cell growth (Shahriyari and Komarova, 2013). Disruptions in AD have been linked to cancerous growth due to unchecked cell division (Hansen and Pelegri, 2021). Specifically for PGLCs, studies indicate they can transform into EGLCs, which share characteristics with PLSCs. These EGLCs demonstrate high SD capabilities and can proliferate through SD under specific *in vitro* conditions (Durcova-Hills et al., 2008). While evidence favors PGCs undergoing AD during development, their capacity for SD *in vitro*, particularly during reprogramming or under specific growth conditions, suggests they may behave similarly to SCs in some contexts (Durcova-Hills et al., 2008). During embryonic development, PGCs follow differentiation pathways that result in the formation of oocytes or spermatogonia. These processes are tightly regulated and typically occur when PGCs transition from SD to AD, producing differentiated offspring. Reprogramming of PGLCs indicates these cells may possess a previously unrecognized level of plasticity; however, this reprogramming does not involve the simultaneous differentiation into less potent cells during their division (Shamblott et al., 1998; Leitch and Smith, 2013).

**FIGURE 2:**
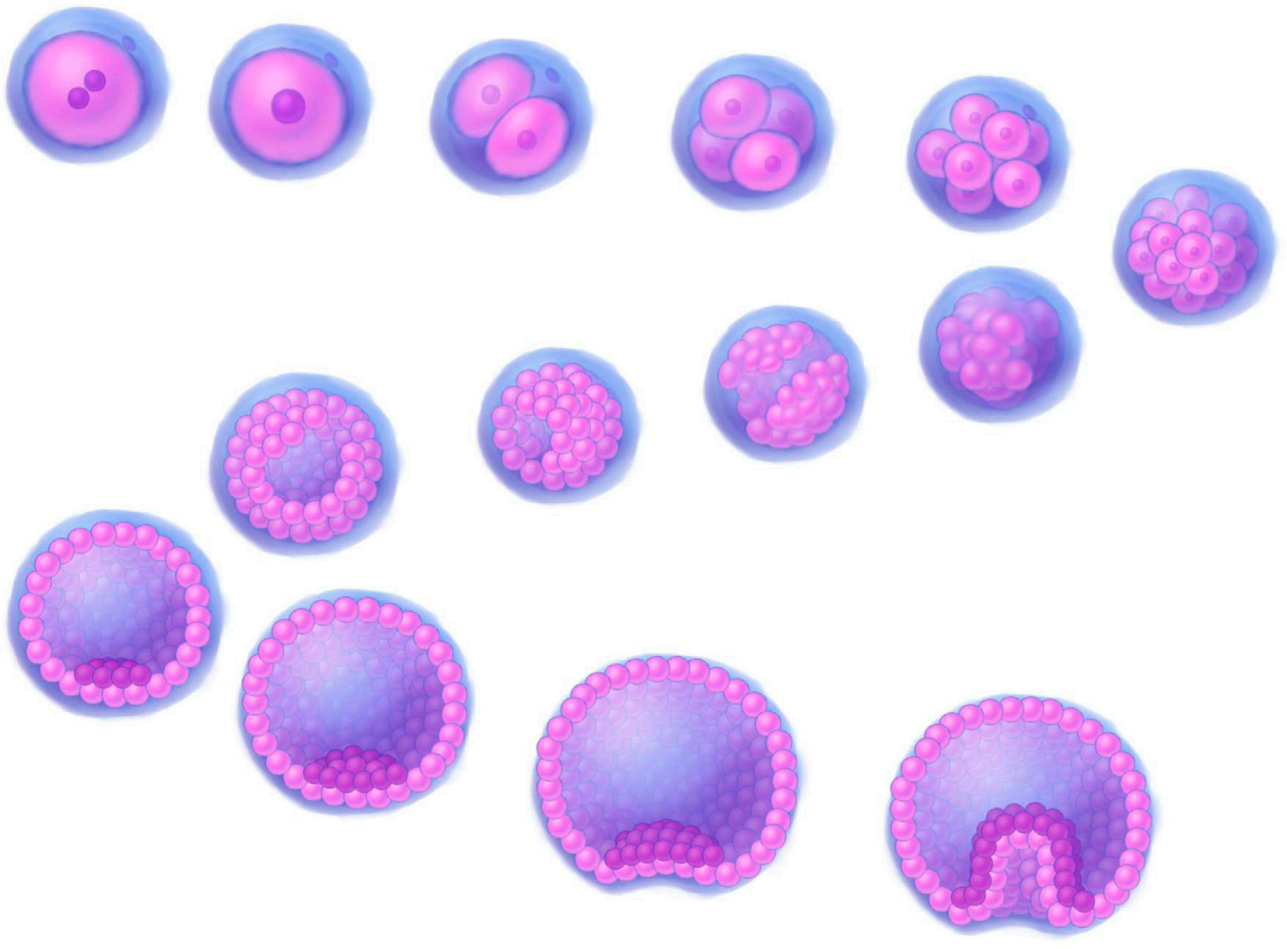
A 3D illustration depicting early stages of human development. Stages of development, progressing downstream from the upper left corner: fertilized egg, zygote, 2-cell stage, 4-cell stage, 8-cell stage, morula (16–32 cells), early blob, late blob, early blastocyst (blastocoel fill <50%), 1_blastocyst (blastocoel fill >50%), 2_blastocyst (blastocoel fill >50%), expanded blastocyst, early gastrula, gastrula. The illustrations were created by graphic artist Mr. Tobiasz Sosnowski, inspired by time-lapse microscopy featured on Iwata et al. (2014) as well on TheDeepSci YouTube channel (accessed 20.11.2024).

Another hypothesis regarding the origin of VSELSCs suggests that they derive from ESCs and serve as precursor cells for tissue progenitors, including PGCs (Ratajczak et al., 2019) (Figure 2). These cells are incorporated into developing tissues as OCT4+ cells. The underlying mechanism involves epigenetic modifications of imprinted genes, such as those at the *Insulin-like growth factor 2*–H19 imprinted maternally expressed transcript (*IGF2–H19*) and *Potassium voltage-gated channel subfamily Q member 1* (*KCNQ1*)–*Cyclin-dependent kinase inhibitor 1C* (*CDKN1C*, formerly known as *p57* or *Kip2*) loci, which maintain these cell stages in a dormant state within adult tissues. A similar process involving the erasure of genomic imprinting also regulates the dormant state of PGCs (Kucia M. et al., 2008; Ratajczak et al., 2019; Ratajczak et al., 2013b).

## Identifying VSELSCs and VSLSLCs: cytological differences from exosomes and cellular debris

Identifying VSELSCs is particularly complex due to their size and morphological similarities to exosomes, extracellular vesicles (EVs), and apoptotic bodies (ABs) (O'Neill et al., 2012). Despite these challenges, both exosomes and VSELSCs play significant roles in various biological processes (Savina et al., 2003; Doeppner et al., 2015; Machtinger et al., 2015; Yáñez-Mó et al., 2015; Zomer et al., 2015; Tkach and Théry, 2016; Bobis-Wozowicz et al., 2017; Kalluri and LeBleu, 2020). However, their unique ability to SD, grow, and proliferate distinguishes VSELSCs from exosomes and other cellular debris (Kucia M. J. et al., 2008; Ratajczak et al., 2019; Domingues et al., 2022).

### EVs

EVs are tiny, membrane-enclosed particles released by cells during various biological activities. Their size and function can vary depending on their cellular origin, influencing their environmental interactions and roles in cell-to-cell communication. EVs transport various molecules, including proteins, lipids, nucleic acids, and signaling compounds, through interactions facilitated by their membranes. Importantly, they are not always completely isolated from the external environment by their lipid membranes. Different EV types include ectosomes which protrude from the cell surface and may assist in intercellular signaling; exosomes, which are approximately 30–150 nm in size and can be identified based on specific membrane markers and proteins; microvesicles, which typically measure 0.1–1 µm in size and are released by cells in response to activation, oxidative stress, or inflammation; and apoptosomes, which vary in size and composition based on the stage of apoptosis and cell type and are surrounded by a lipid membrane containing cellular fragments such as nuclear debris or organelles (Kowal et al., 2016; Muhsin-Sharafaldine and McLellan, 2018; Théry et al., 2018; Van Niel et al., 2018; Jeppesen et al., 2019; Crescitelli et al., 2021).

The structural similarities between these EV types and ABs suggest that they could be classified as forms of ABs; conversely, ABs might also be classified as EVs (Table 2). Currently, no specific antibodies target EVs. The presence of specific surface markers can vary depending on the cell type, differentiation stage, physiological processes, microenvironment, and other factors, limiting their specificity. However, some antibodies target surface proteins present on EVs, which can aid their identification and characterization. Common markers and antibodies used in EV studies include tetraspanins (CD9, CD63, CD81), surface markers associated with programmed cell death 6 interacting protein (PDCD6IP) and tumor susceptibility 101 (TSG101), as well as proteins such as heat shock proteins: HSP70, HSP90; and flotillin 1 (FLOT1) (Bobrie et al., 2012; Andreu and Yáñez-Mó, 2014; Li et al., 2017; Merchant et al., 2017; Li et al., 2019; Moeinzadeh et al., 2022; Zhang et al., 2022; Kalluri and McAndrews, 2023).

**TABLE 2:** Cytological characteristics and comparison of the VSELSCs, VSLSLCs, and SLSLCs with ABs and EVs.

Event type					
Cytological parameter	VSELSC	VSLSLC	SLSLC	AB	EV
Median diameter (µm)	Around 5–7	Around 5–7	Around 7–9	1–5	0.1–1
Membrane integrity	Yes	Yes	Yes	Unusual	Unusual
Specific cell surface markers	Yes	Yes	Yes	Unusual	Unusual
Cytoplasm pH	Deeply basophilic	Deeply basophilic	Deeply basophilic	Unusual	Not specific
DNA	Yes	Yes	Yes	Yes	Unusual
DNA condensation	High	High	High	Very high	Not specific
Cell fusion/absorption	Yes	Yes	Yes	Yes	Yes
Symmetric division	Yes	Yes	Yes	No	No
Specific surface markers	SSEA4^+^ OCT4^+^ CD34^+^ CD133^+^ CXCR4^+^ CD45^−^ CD2^−^ CD56^−^ CD38^−^	CD34^−^ CD38^−^ CD45^−^ CD56^−^ CD2^−^	CD34^+^ CD38^−^ CD45^−^ CD56^−^ CD2^−^	CD14^+^ CD24^+^ CD31^+^ CD34^+^ CD44^+^	CD9^+^ CD63^+^ CD81^+^

Abbreviations: VSELSCs, very small embryonic-like stem cells; VSLSLCs, very small leukemic stem-like cells; SLSLCs, small leukemic stem-like cells; ABs, apoptotic bodies; EVs, extracellular vesicles. We identified CD38 expression in VSLSLCs and SLSLCs as one of the key markers for the primary population of LSLCs (Lica et al., 2021).

### ABs

ABs are typically small, circular, or oval structures, ranging from 1 to 5 µm in diameter, formed during apoptosis, the programmed cell death process. The cell’s chromatin undergoes significant condensation during apoptosis (Lecoeur et al., 1997; Coleman et al., 2001; Zhang et al., 2018; Aoki et al., 2020; Dou et al., 2020; Serrano-Heras et al., 2020). As the process advances, the cell shrinks, and its fragmented components are encapsulated within vesicles, which may be surrounded by a double membrane, which provides some protection to the contents but does not ensure complete integrity (Kerr et al., 1972; Lecoeur et al., 1997; Coleman et al., 2001; Zembruski et al., 2012; Zhang et al., 2018; Aoki et al., 2020; Dou et al., 2020; Serrano-Heras et al., 2020). Early-stage ABs may not exhibit positive staining for 7-aminoactinomycin D (7-AAD), indicating complete isolation (Montgomery et al., 2020). Importantly, this study did not cover VSELSCs and their morphology. Furthermore, the outer membrane of ABs can display specific CD surface markers, although this is not consistent across all ABs (Kerr et al., 1972; Lecoeur et al., 1997; Coleman et al., 2001; Zhang et al., 2018; Aoki et al., 2020; Dou et al., 2020; Serrano-Heras et al., 2020). The presence of these markers varies depending on the cell type (including the primitive SCs), apoptosis stage, and other factors, making them characteristics with limited specificity.

While exosomes and ABs are distinct entities, they share several features (Table 2). Research on exosomes has often identified common markers such as tetraspanins (e.g., CD63 and CD81), which are also found on ABs (Yáñez-Mó et al., 2015). The complement system is crucial for recognizing and eliminating ABs. The complement protein complex C1q binds to phosphatidylserine and other molecules on AB surfaces, facilitating their clearance by phagocytes and helping to prevent autoimmunity (Païdassi et al., 2008). During apoptosis, DNA and chromatin break apart, exposing histones on the AB surface, which serve as recognition markers (Zernecke et al., 2009). ABs frequently change their membrane protein composition, often losing membrane integrity. Markers associated with ABs include altered membrane proteins such as death receptors (e.g., Fas cell surface death receptor) and apoptosis-regulating proteins from the BCL2 apoptosis regulator family (Nagata, 2018). The tetraspanin CD9 is involved in various cell processes such as adhesion and membrane fusion, and its presence on ABs may enhance cell communication and recognition (Miyanishi et al., 2007). CD14, which binds intercellular adhesion molecule 3 (ICAM3), facilitates interactions between ABs and macrophages for recognition and clearance (Moffatt et al., 1999; Hart et al., 2008). ABs can also express integrins or other adhesion molecules that aid in phagocytic interactions. Exposed on AB surfaces, ICAM3 plays a key role in their recognition by phagocytes (Devitt et al., 2003; Hart et al., 2008). Various lectins that recognize altered sugars on cell membranes and CD14 are important for recognizing and clearing ABs. CD24, associated with cell adhesion and immune modulation, may also facilitate interactions between ABs and immune cells (Liu et al., 2002; Devitt et al., 2003). CD36, a scavenger receptor, promotes AB phagocytosis by recognizing phosphatidylserine on its surface (Greenberg et al., 2006). Platelet endothelial cell adhesion molecule (PECAM1) also known as cluster of differentiation 31 (CD31), another adhesion protein, signals phagocytes when its presence decreases on AB surfaces, marking them for removal (Brown and Savill, 1999). Additionally, the adhesion molecule CD44, which is involved in cell interaction and migration, may contribute to phagocytosis when exposed on ABs (Poon et al., 2014). CD47 typically serves as a “do not eat me” signal for macrophages, but its expression decreases during apoptosis, allowing ABs to be recognized and cleared by the immune system (Gardai et al., 2005). Recent research has highlighted key surface markers on human ABs, particularly phosphatidylserine, that signal phagocytes. Receptors from the TIM and TAM families (hepatitis A virus cellular receptor 1 [HAVCR1], T cell immunoglobulin and mucin domain containing 4 [TIMD4], MER proto-oncogene, tyrosine kinase [MERTK], TYRO3 protein tyrosine kinase [TYRO3], and AXL receptor tyrosine kinase [AXL]) recognize this marker, facilitating the removal of apoptotic cells and regulating inflammation, particularly in cancer and autoimmune diseases (Fadok et al., 2000; Serrano-Heras et al., 2020; Yu et al., 2023; Kur and Weigert, 2024; Lica et al., 2024). Isolating ABs from patient blood samples may offer valuable diagnostic and therapeutic insights, especially for neurological conditions such as stroke and neurodegenerative diseases (Serrano-Heras et al., 2020).

### VSELSCs

Human VSELSCs are small cellular structures, typically measuring 5–7 µm in diameter, and exhibit unique embryonic or multipotent characteristics, including the ability to SD and differentiate into various cell types (Sovalat et al., 2011; Shin et al., 2012; Vojnits et al., 2014; Kakavoulia, 2021). While the concept of VSELSCs is promising, further research is needed to elucidate their biological mechanisms and improve the accuracy of detection methods (Abbott, 2013; Nicholls, 2013; Szade et al., 2013; Dulak et al., 2015). Despite these challenges, an increasing number of independent studies support the existence of VSELSCs. Cellular debris, comprising fragments resulting from cellular disintegration, apoptosis, or other cellular activities, can be mistakenly identified as living cells during flow cytometry analysis. Data on global gene expression in murine VSELSCs indicate that key pluripotency markers such as *Pou5f1*, *Nanog*, and *SRY-box transcription factor 9* [*Sox2*], along with the involvement of Polycomb proteins, play essential roles in maintaining the pluripotent characteristics of these cell stages (Shin et al., 2012).

### VSLSLCs

Our earlier studies (Lica et al., 2018, 2021; Lica and Pradhan, 2023) revealed a phenomenon previously observed in healthy human HSCs: the enrichment of culture with primitive stages through cell density control (Csaszar et al., 2012). The LSLCs we identified exhibited an average size of approximately 9–12 µm (Lica et al., 2018, 2021).

Potential VSLSLCs were expected to have a similar size to VSELSCs, LT-LSCs and other small progenitor/precursor SCs (PSCs), which may increase during developmental stage transformations, potentially reaching approximately twice the size during SD or when forming LSLCs (Lica et al., 2018; Lica et al., 2021; Lica and Pradhan, 2023). The corresponding morphological features of these stage transformations should be visible in scatter dot plots, representing the resting phase (around 5–7 µm), SD (up to 10–14 μm, although it might also be 5–7 µm), and AD (up to 13–16 µm), leading to an increase in size through developmental stages. We cannot exclude the occurrence of other ADs at cell sizes of 5–7 μm, which may lead to the formation of smaller, less differentiated cells compared to LSLCs. Given the morphological similarities among hypothetical developmental stages, we have divided the scatter dot plot’s region of interest into two distinct events: VSLSLCs and small LSLCs (SLSLCs). Notably, SLSLCs may represent a developmental stage of VSLSLCs undergoing by SD or AD changes in DNA distribution. Identifying specific CD markers that uniquely target SCs, CSCs, LSCs, and their precursors is challenging (Rix et al., 2022). Due to their morphology, VSLSLCs can be mistaken for EVs and ABs in some assays and *vice versa*. The involvement of EVs and ABs in cellular development is an area of intense research, as many studies indicate (Denzer et al., 2000; Migneault et al., 2020; Phan et al., 2020; Beltraminelli et al., 2021; Gregory and Rimmer, 2023).

Very small cellular debris and EVs generally appear in scatter dot plot regions that are difficult to quantify. EVs and ABs may rarely mimic VSLSLCs, as they are a few micrometers in size and can contain strong condensed DNA fragments, similar to cell nuclei, surrounded by a double membrane with surface markers. They are differentiated from VSLSLCs by their round membranes, alkaline pH environment, and immature, less condensed chromatin. The 7-AAD dye aids in distinguishing them on scatter dot plots from entities with intact membranes. However, since some EVs and ABs are similar in size and content and can display CD markers, they may not allow the entry of 7-AAD and similar dyes in early stages, complicating their differentiation from (very) small cell developmental stages. We hypothesize that VSLSLCs function as precursors to LSLCs, and their numbers in cell density-dependent different leukemic sublines, along with other developmental stages, should exhibit significant differences (Lica et al., 2018; Lica et al., 2021). Considering the data regarding CD markers for VSELSCs, we propose that hematopoietic progenitor cell antigen CD34 could be one of the first markers observed on the cell surface. For the negative marker for VSELSCs, we selected receptor-type tyrosine-protein phosphatase C (CD45 antigen) since it is commonly used by researchers (Lacombe et al., 1997; Kuruca et al., 2019; Filidou et al., 2023) and is part of the EuroFlow standardization for hematological probes (Van Dongen et al., 2012; Glier et al., 2019). CD markers that exclusively target VSELSCs, such as NANOG and CD44, stage-specific embryonic antigen-4 (SSEA4), CD133 antigen, show lower efficiency in cell lines (Gunjal et al., 2015; Sellers et al., 2017) than in samples directly from organisms and CD44 antigen can be present on the surface of apoptotic bodies, potentially invalidating and distorting results by causing false positives or negatives. To ensure consistency in interpreting findings from previous studies (Lica et al., 2018; Lica et al., 2021) and to distinguish between different developmental stages within the tested cell lines, we chose neural cell adhesion molecule (CD56 antigen) as an indicator of a poorer prognosis in multiple myeloma (Lanier et al., 1989; Zheng et al., 2022). To confirm the presence of DNA and its degree of condensation, we examined the CD34 marker level relative to DNA content.

Briefly, VSLSLCs can directly increase their numbers and indirectly enhance the number of lower hierarchical developmental cell stages in culture. They are likely to exhibit a triple-negative status (CD45^−^/CD56^−^/CD2^−^) for the tested CD markers and a positive status for CD34, with morphology exhibiting alkaline cytoplasm and less condensed DNA compared to ABs while being negative for 7-AAD. However, cytological analysis indicates that the populations of the most primitive cells can be divided into CD34^−^ VSLSLCs and CD34^+^ SLSLCs (Lica and Pradhan, 2023). Notably, CD34 is a surface protein commonly associated with HSCs and is used as a marker in various leukemia types, including acute lymphoblastic leukemia (ALL) and AML. However, studies show that a significant subset of patients with ALL and AML do not express CD34 on leukemic cells (Aljurf et al., 2011; Ho et al., 2020; Araki et al., 2023; Araki et al., 2024). Research has demonstrated that CD34^−^ cells can still exhibit SC characteristics, especially among leukemic populations, indicating that CD34 is not essential for identifying or isolating LSCs *in vitro* (Lica and Pradhan, 2023).

CD34 is also not characteristic of mesodermally specified embryoid bodies that form HPCs (Bauwens et al., 2008; Demirci et al., 2020; Anjos-Afonso and Bonnet, 2023). Mesodermally specified embryoid bodies typically range in size from approximately 150–450 μm, depending on culture conditions and developmental stage. Their size significantly influences their differentiation potential, with smaller bodies favoring endodermal differentiation and larger ones supporting mesodermal development. Controlling their size is a crucial factor in optimizing differentiation protocols for specific lineages (Bauwens et al., 2008; Demirci et al., 2020).

Given the morphological similarities among these structures, we provide an overview of their distinct characteristics in Table 2.

For more detailed information, please refer to our previous work (Lica et al., 2018; Lica et al., 2021; Lica and Pradhan, 2023).

## Cytological characteristics of small stem progenitors in embryos and adults

### EpibCs

In humans, these cells typically measure 10–15 µm in size. While their size may vary slightly depending on the cell cycle phase, it generally falls within this range. At the epiblast stage, the cells are pluripotent, meaning they can develop into any cell type in the body, including those from the ectoderm, mesoderm, and endoderm (Figure 1). ESCs (EpibC precursors) appear at an early stage of development and are extracted from the ICM of the embryo during the blastocyst phase before the embryo implants in the uterus. The blastocyst forms around 5–6 days after fertilization (Figure 2); at this stage, the embryo consists of two main groups: the ICM, which will develop into the fetus, and the trophectoderm, which surrounds the ICM and contributes to forming structures like the placenta (Thomson et al., 1998).

### MdPCs

In adult humans, these cells are similar in size to other types of progenitor SCs, typically measuring around 10–15 µm. Their size varies based on their developmental and differentiation stage (Asahara et al., 1997; Erices et al., 2000; Igura et al., 2004; Kögler et al., 2004; Zhang et al., 2011; Divya et al., 2012; Lyahyai et al., 2012; Slukvin and Kumar, 2018). As these cells specialize into specific cell types, such as muscle, bone, or blood cells, their dimensions may adjust to suit the requirements of their final function. They can differentiate into mesenchymal and endodermal lineages, although their pluripotency gene expression is limited (Figure 1). These cells exhibit regenerative potential, like VSELSCs, and express surface markers such as endoglin (CD105), 5′-nucleotidase (CD73), and Thy-1 membrane glycoprotein (CD90) but not CD45, aligning with their role in tissue regeneration (Asahara et al., 1997; Erices et al., 2000; Igura et al., 2004; Kögler et al., 2004; Zhang et al., 2011; Divya et al., 2012; Lyahyai et al., 2012; Slukvin and Kumar, 2018).

### EnthPCs

These cells measure 10–15 μm, primarily differentiate into endothelial cells, and have limited potential for forming other cell types (Figure 1). They play a crucial role in vascular regeneration and share molecular similarities with VSELSCs, especially regarding their regenerative capabilities. EnthPCs are characterized by the expression of markers such as CD34, vascular endothelial growth factor receptor 2 (VEGFR2), CD133 antigen, and other markers that are associated with endothelial cells (Aprile et al., 2024).

### MsPCs

These cells can differentiate into various specialized cells, including osteocytes (bone cells), chondrocytes (cartilage cells), and adipocytes (fat cells) (Figure 1). MsPCs can be isolated from diverse tissues, including bone marrow, adipose tissue, skeletal muscle, and umbilical cord tissue (Reyes et al., 2001; Kögler et al., 2004; Ulloa-Montoya et al., 2007; Kuroda et al., 2010; Phermthai et al., 2010; Demerdash et al., 2020; Chamling et al., 2021; Budeus et al., 2023; Ghabriel et al., 2023; Ebrahim et al., 2024). Typically, MsPCs measure around 15–30 µm in size, although their dimensions can change based on the culture environment, and reports indicate that they may shrink to as small as 6–8 µm (Han et al., 2019; Qu et al., 2024). The key molecular signaling pathways for MsPCs include BMP, which is essential for the differentiation into osteocytes and chondrocytes; Wnt/β-catenin, which regulates cell proliferation and differentiation; NOTCH, which is important for SD and differentiation, particularly towards muscle cell formation; the phosphatidylinositol 3-kinase (PI3K)/Akt which governs cell survival, proliferation, and differentiation; and Hippo-transcriptional co-activators Yes-associated protein (YAP)/transcriptional co-activator with PDZ-binding motif (TAZ), which regulates cell growth, differentiation, and response to mechanical stress. MsPCs are characterized by specific positive surface markers such as CD105^+^, CD73^+^, CD90^+^, and CD44^+^ while lacking hematopoietic markers CD11b^−^, CD14^−^, CD19^−^, CD34^−^, CD45^−^, CD79a^−^, or HLA class II histocompatibility antigen, DR alpha chain (HLA-DR^−^) and pluripotency markers such as OCT4 and SSEA4 (Pittenger et al., 1999; Caplan, 2005; Dominici et al., 2006; Phinney and Prockop, 2007; Uccelli et al., 2008).

### Long-term HSCs (LT-HSCs)

LT-HSCs are small, comparable in size to small lymphocytes, with a typical diameter of 7–10 µm (Figure 1). They have the capacity for long-term SD and can function throughout an organism’s lifetime. LT-HSCs maintain a distinct epigenetic program characterized by a closed chromatin state, which preserves gene silencing and prevents unwanted differentiation. Their low metabolic activity and slow cell cycle mean they can sustain their SD ability for extended periods (Wang and Ema, 2016). These cells are quiescent, entering dormancy to protect against environmental stresses, including oxidative damage. They rely on glycolytic metabolism, which lowers oxygen consumption and minimizes oxidative stress. LT-HSCs express genes such as *forkhead box O1* (*FOXO1), transforming growth factor beta 1 (TGFB1) and NFE2 like bZIP transcription factor 2 (NFE2L2)*, which are involved in maintaining quiescence and defending against oxidative stress. Autophagy plays a critical role in clearing damaged organelles. Compared to short-term HSCs (ST-HSCs), LT-HSCs exhibit higher activity in stress-protective pathways, including TGFβ, p53, and FoxO, which enhance their long-term SD potential. The Wnt pathway is also more active in LT-HSCs, helping regulate their regenerative capacity. Additionally, LT-HSCs exhibit a more closed chromatin state, further preserving gene silencing and preventing premature differentiation. Their higher expression of oxidative stress protection genes equips LT-HSCs to tolerate damage that could lead to cellular degeneration. Conversely, ST-HSCs are more susceptible to damage but are more efficient in short-term blood cell production (Wilson et al., 2007). Notably, human LT-HSCs and ST-HSCs express slightly different surface markers than their mouse counterparts, particularly regarding CD34; human LT-HSCs are CD34^+^, while mouse LT-HSCs are CD34^−^. Other markers, such as CD38, CD90, CD45RA, and CD49f, help distinguish LT-HSCs from ST-HSCs. Interestingly, certain conditions may give rise to a CD34^−^ LT-HSC population in humans, which may support long-term renewal and multipotency (Notta et al., 2011; Vedi et al., 2016; Ho et al., 2020).

### ST-HSCs

ST-HSCs vary in size depending on their cell cycle stage, with diameters ranging from 8 to 12 µm. They have limited SD capacity and are more metabolically active than LT-HSCs. ST-HSCs primarily participate in the rapid replenishment of blood cells and are crucial for short-term regenerative functions. Initially believed to support hematopoiesis for only 4–12 weeks, recent studies indicate their capacity may extend beyond this period. Experimental models have shown ST-HSCs can maintain engraftment for up to 1 year in primary recipients and at least 3 months in secondary recipients, suggesting a broader long-term potential than previously assumed; however, their overall engraftment ability remains lower than that of LT-HSCs. Despite their restricted SD ability, ST-HSCs predominantly differentiate HSCs/multipotent progenitor cells (MPPs), rapidly replenishing blood cell populations. They enter the cell cycle more quickly than LT-HSCs, facilitating faster differentiation. Key signaling pathways such as Janus kinase (JAK) signal transducer and activator of transcription (JAK-STAT) (promoting proliferation and differentiation), mammalian target of rapamycin (mTOR) (whose excessive activation can deplete SD), and mitogen-activated protein kinase (MAPK)/extracellular signal-regulated kinase (ERK) (MAPK/ERK) (involved in proliferation and differentiation) are more active in ST-HSCs. Compared to LT-HSCs, ST-HSCs have a more “open” chromatin state, increasing transcriptional accessibility to genes related to differentiation and proliferation. Genetictened mitotic activity renders them more susceptible to genetic damage and oxidative stress, particularly from reactive oxygen species, due to their reliance on oxidative phosphorylation. The increased activity of proliferation and differentiation pathways drives ST-HSCs to differentiate more rapidly, though this acceleration limits their regenerative potential. Unlike LT-HSCs, ST-HSCs express surface markers such as CD34 and CD38 but not CD90, correlating with their rapid differentiation capacity (Wilson et al., 2007; Cabezas-Wallscheid et al., 2014; Zeng et al., 2022).

## Other primitive adult PSCs

Several additional PSCs in the adult human body are similar in size, regenerative capacity, and differentiation potential to VSELSCs (Bhartiya et al., 2022).

### Marrow-isolated adult multilineage inducible cells (MIAMICs)

These cells are a unique population derived from human bone marrow, typically 6–12 µm in size (D’Ippolito et al., 2004) (Figure 1). They are a specialized subset of MsPCs that, under appropriate *in vitro* conditions, can differentiate into various cell types, including osteoblasts, chondrocytes, adipocytes, and even nerve cells. MIAMICs express key pluripotency genes, such as *POU5F1P5* (synonym *OCT4*), *SOX2*, and *NANOG*, albeit at lower levels than ESCs or iPSCs. Notably, MIAMICs can survive in challenging environments, including hypoxia, indicating a resilience not commonly seen in other adult SCs. Their expression of *SSEA4* and other pluripotency genes, coupled with their stress resistance, suggests they may have enhanced flexibility in differentiation. MIAMICs are characterized by the absence of hematopoietic markers (CD34^−^ CD45^−^) and the presence of CD29^+^, CD73^+^, CD90^+^, CD105^+^, and SSEA4^+^, a marker used to identify pluripotent cells, including ESCs (D’Ippolito et al., 2004; Rossi et al., 2019).

### Multilineage-differentiating stress enduring cells (MUSECs)

MUSECs are SCs isolated from human bone marrow and various other tissues, generally measuring 10–15 µm in diameter (Kögler et al., 2004; Wakao et al., 2011) (Figure 1). These cells can differentiate into multiple lineages derived from all three germ layers: mesoderm, ectoderm, and endoderm. Their differentiation potential is linked to the expression of key pluripotency genes (Wakao et al., 2011, 2012; Leng et al., 2019). MUSECs express surface markers such as stage-specific embryonic antigen 3 (SSEA3), commonly found in PLSCs, and CD105, which is also a marker for MsPCs. However, they do not express CD90, CD34, or CD45. Their unique molecular characteristics, including the expression of pluripotency genes (*OCT3*, *OCT4*, *SOX2*, *NANOG*) and their ability to withstand stress and retain genomic stability make them promising candidates for regenerative therapies (Wakao et al., 2011).

### CD133^+^ PSCs

CD133^+^ PSCs have been identified in various human adult tissues, including peripheral blood, bone marrow, the brain, and even in cancerous tissues (Shmelkov et al., 2008; Wu and Wu, 2009; Kreso and Dick, 2014; Shaikh et al., 2015; Glumac and LeBeau, 2018; Wang et al., 2020; Korn et al., 2021; Nayak et al., 2022; Liu et al., 2023). Their median diameter is approximately 5–10 μm, typical for small, primitive hematopoietic cells (Figure 1). This size variation may indicate adaptation to specific microenvironments and potentially reflect a more primitive or specialized function within the hematopoietic system (Hawley et al., 2006; Mizrak et al., 2008). CD133^+^ PSCs express markers characteristic of various PSC types, such as those of endothelial, hematopoietic, and neurogenic origins. However, unlike VSELSCs, CD133^+^ PSCs express lower levels of pluripotency markers, indicating a more specialized state. Importantly, CD133 is not exclusive to SCs; it is also found in various types of healthy cells and MCs (Kreso and Dick, 2014; Korn et al., 2021; Liu and Qian, 2021; Nayak et al., 2022). CD133 has gained attention as a CSC marker in cancer research, suggesting its involvement in tumor formation and resistance to cancer therapies. The potential use of CD133^+^ PSCs in regenerative medicine, particularly for heart tissue repair, vascular regeneration, and neurogenesis, is an area of growing interest. However, the role of CD133 as a definitive marker for precursor and progenitor SCs remains debated.

### Node and duct SCs (NDSCs)

NDSCs are SCs derived from nodes and ducts within the body, primarily associated with hematopoietic and neural systems (Lee et al., 2014). Present in the vascular systems, these cells can differentiate into neuronal cells *in vitro*. In an *in vivo* model of ischemic brain injury, NDSCs exhibited promising regenerative effects, highlighting their proliferative potential (Lee et al., 2014). Typically ranging from 6 to 8 µm in diameter, NDSCs share characteristics with both HSCs and neural SCs, given their ability to differentiate into both blood and neuronal cells. They are relatively small, with sizes comparable to LT-HSCs (Figure 1). Their small size enables them to effectively circulate through the vascular system, which is crucial for their role in tissue repair and regeneration. NDSCs are regulated by several key molecular pathways during differentiation, including the BMP, fibroblast growth factor (FGF), and retinoic acid (RA). They exhibit a distinct immunophenotype, often expressing key surface markers such as CD34 (commonly used to identify SCs, particularly HSCs and MsPCs), CD73 (indicating immunomodulatory capacity and support for regenerative processes), CD90^+^ (associated with tissue repair and regenerative potential), and CD44 (involved in cell adhesion to the extracellular matrix and SC mobility, playing a role in tissue repair) but not Lin (lineage negative, indicating the absence of markers typical of mature, committed cells) and CD45 (characteristic of mature immune and blood cells). These markers are essential for identifying and isolating NDSCs from other SC types. NDSCs activate several signaling pathways that regulate their proliferation, differentiation, and responses to environmental cues: Wnt/β-catenin regulates SD and enhances proliferation; NOTCH controls differentiation and helps maintain the SC state; TGF-β plays a role in maintaining SC homeostasis by regulating the transition from quiescence to active differentiation and influencing the immunomodulatory abilities of these cells; PI3K/AKT/mTOR controls proliferation and survival (mTOR overactivation may reduce regenerative capacity, but modulating this pathway supports their repair functions); and hypoxia-inducible factors (HIF) facilitates adaptation and function in low-oxygen niches, promoting survival and maintaining pluripotency in stressful, oxidative conditions. These pathways guide the cells through processes that lead to specific lineage commitments depending on environmental cues and specific growth factors. Research into the therapeutic application of NDSCs has revealed their potential for treating neurological disorders, autoimmune diseases, and cancer by targeting damaged tissues and supporting cellular regeneration (Lee et al., 2014).

### Spore-like cells (SpLCs)

SpLCs represent a relatively new and intriguing area of research in humans. These cells are thought to be resilient to extreme environmental conditions, similar to bacterial spores. Studies suggest that SpLCs might play a role in regeneration, stress survival, and potential cancer resistance (Kajstura et al., 2011). Research into SpLCs also highlights their potential for tissue regeneration (D’Amour et al., 2005; Shmilovici, 2007; Rahimi Darehbagh et al., 2024). Their stress-endurance capabilities, including the expression of stress-related genes (e.g., *hypoxia inducible factor 1 subunit alpha gene: HIF1A*) and autophagy markers (e.g., LC3), enable them to withstand adverse conditions and reactivate when the environment improves (Li and Clevers, 2010). Research into CSCs suggests that these resistant cell types could be crucial for developing therapies to prevent cancer relapse by targeting dormant cancer cells. They are being studied not only in cancer but also in neurodegenerative diseases such as Alzheimer’s and Parkinson’s, where they may contribute to cellular survival under oxidative stress (Cui et al., 2024). The molecular mechanisms by which SpLCs differentiate into mesodermal lineages involve several key processes guided by the Wnt and BMP pathways, which promote mesodermal development by inducing mesoderm-specific genes (Vacanti et al., 2001). Activation of these pathways promotes the early stages of mesodermal development. Key transcription factors such as T-box transcription factor T (TBXT), Mesoderm posterior protein 1 (MESP1), and Transcription factor GATA-4 are upregulated during mesodermal differentiation (Vacanti et al., 2001), orchestrating the transition of pluripotent cells into mesodermal progenitors that can give rise to muscle, bone, and cardiovascular system. Epigenetic modifications are vital in silencing pluripotency markers (e.g., OCT4 and NANOG) and activating mesoderm-specific genes, ensuring commitment to the mesodermal lineage (Vacanti et al., 2001). The concept of SpLCs overlaps with findings about VSELSCs and MUSECs, which are also recognized for their ability to survive under stress and potentially regenerate tissues and malignancies research (Figure 1) (D’Amour et al., 2005; Shmilovici, 2007; Rahimi Darehbagh et al., 2024).

## Mechanistic comparison of human adult very small and small SC progenitors

### MUSECs, MIAMICs and NDSCs vs. LT-HSCs

MUSECs and MIAMICs exhibit strong resistance to stress, expressing key genes involved in autophagy and regeneration. However, unlike MIAMICs and LT-HSCs, which require specific environmental conditions for differentiation, MUSECs can spontaneously differentiate when exposed to stress. This ability sets them apart. MUSECs express pluripotency markers, such as OCT4 and SSEA3, which gives them broader potential for differentiation into various cell types compared to LT-HSCs. While MUSECs express similar multilineage differentiation markers to MIAMICs, they are notable due to their higher proliferative capacity (Alanazi et al., 2023). In contrast, like MIAMICs and LT-HSCs, NDSCs can regenerate but seem more limited in their differentiation potential, primarily focusing on specific tissues such as ducts and lymph nodes. The unique gene expression profile of NDSCs, tied to their specialized regenerative role in these structures, differentiates them from the more versatile MIAMICs (Urrutia et al., 2019; Alanazi et al., 2023).

### VSELSCs vs. MIAMICs and LT-HSCs

VSELSCs and MIAMICs share several characteristics with LT-HSCs, particularly long-term regenerative capacity, ability to remain quiescent, and resistance to oxidative stress. Both VSELSCs and MIAMICs resemble LT-HSCs in maintaining homeostasis and promoting cellular longevity. Like LT-HSCs, VSELSCs are quiescent cells that remain in a low metabolic state to protect themselves from oxidative stress and DNA damage. VSELSCs and LT-HSCs both possess long-term SD capabilities, becoming active under specific conditions, such as during tissue repair. However, unlike VSELSCs, LT-HSCs do not express some ESC markers, such as SSEA4 and OCT4, suggesting pluripotency. VSELSCs also demonstrate enhanced activity in pathways promoting cellular quiescence and stress resistance, including FoxO and TGF-β, which are characteristics shared with LT-HSCs (Wojakowski et al., 2008; Ratajczak et al., 2012). Compared to VSELSCs, MIAMICs and LT-HSCs both exhibit higher expression of genes involved in immunoregulation, as well as low proliferative activity. Both MIAMICs and LT-HSCs express low levels of markers associated with active cell cycling, contributing to their long-term survival and tissue regeneration. Studies have demonstrated that MIAMICs can maintain a quiescent phase with minimal expression of cell cycle markers, much like LT-HSCs, which also remain inactive for extended periods (D’Ippolito et al., 2004). This dormancy helps LT-HSCs preserve their regenerative potential and avoid early depletion. Like LT-HSCs, MIAMICs rely on glycolytic metabolism, enabling them to function efficiently in oxygen-poor environments. This metabolic strategy helps limit oxidative damage, a trait they share with LT-HSCs (Beyer Nardi and Da Silva Meirelles, 2006). Furthermore, MIAMICs express higher levels of genes linked to immune regulation and autophagy, like LT-HSCs, supporting their regenerative functions. Key genes in this process include *Forkhead box O1 (FOXO1), Transforming growth factor beta 1 (TGFB1), and autophagy-related genes such as Autophagy related 5 (ATG5) and Beclin 1 (BECN1)*, which are critical in LT-HSC biology. Compared to VSELSCs, MIAMICs exhibit a glycolytic metabolic profile similar to LT-HSCs, although they differ in specific signaling pathways. For example, MIAMICs are more active in pathways related to mesenchymal differentiation, while LT-HSCs are more specialized toward hematopoiesis (Wojakowski et al., 2008; Ratajczak et al., 2012; Filippi and Ghaffari, 2019).

### VSELSCs vs. MUSECs and MIAMICs

MUSECs express some pluripotency markers, such as OCT4, but their expression is weaker than that of VSELSCs. MUSECs also demonstrate greater resistance to stress than VSELSCs. Similarly, MIAMICs exhibit low expression of pluripotency markers, with their differentiation potential being more restricted to mesenchymal lineages. On a molecular and immunophenotypic level, MUSECs and MIAMICs are most similar to VSELSCs due to their shared expression of pluripotency markers and stress resistance. However, VSELSCs have a broader differentiation potential, more like ECs. In contrast, LT-HSCs and USSCs are more specialized towards specific lineages (hematopoietic and mesenchymal, respectively), making them less comparable to VSELSCs in terms of versatility (Ratajczak et al., 2012; Alanazi et al., 2023; Velasco et al., 2023).

### VSELSCs vs. SpLCs and CD133^+^ PSCs

Both SpLCs and CD133^+^ PSCs exhibit appreciable similarities to VSELSCs, particularly regarding their diameter (VSELSCs: 5–7 μm, SpLCs: 3–5 μm, very small CD133^+^ PSCs: 5–10 μm) and especially in the case of CD133^+^ PSCs expression of pluripotency markers (e.g., OCT4, NANOG, SOX2 and SSEA3). All these cells stages can play significant roles in the regenerative processes of adult tissues (Alanazi et al., 2023; Velasco et al., 2023).

## Cellular plasticity: transdifferentiating or VSELSCs

Ongoing research into cellular plasticity has revealed that SCs may be able to shift their differentiation path. This phenomenon could result from transdifferentiation, where differentiated cells transform into other cell types, or it might be attributed to the presence of early-stage SCs in adult tissues (Graf and Enver, 2009; Jopling et al., 2011; Ratajczak et al., 2013b). Transdifferentiation is the process by which differentiated cells, under specific conditions, change their fate and become another cell type without reverting to an SC stage. This process typically requires induction through transcription factors and genetic modifications (Jopling et al., 2011). Notable factors that induce transdifferentiation include Myoblast determination protein 1 (MyoD1), which can convert fibroblasts into muscle cells, and Pancreas/duodenum homeobox protein 1 (PDX1), which can transform pancreatic alpha cells into insulin-producing beta cells. Additionally, factors such as OCT4, SOX2, KLF4, and c-MYC, which are used to generate iPSCs, also facilitate transdifferentiation by altering cell fates (Blau and Daley, 2019).

An alternative theory posits that adult tissues harbor PLSCs, specifically VSELCSs, which are responsible for cellular plasticity rather than transdifferentiated cells (Ratajczak et al., 2013b). According to their discoverers, VSELCSs reside in a dormant state within adult tissues, and this dormancy is regulated by epigenetic modifications in genes associated with insulin signaling pathways (Ratajczak et al., 2013a). In regenerative medicine, VSELSCs are considered valuable because they can differentiate into various types of adult cells, and unlike iPSCs, they do not appear to carry oncogenic risks (Jiang et al., 2002; Cao et al., 2006; Masuda, 2012).

The origins of VSELSCs remain debated. If VSELSCs are indeed responsible for cellular plasticity and originate early in embryonic development, they may derive from either EpibCs or PGCs (Park et al., 2009). Their ability to exhibit pluripotency similar to iPSCs suggests that they have a higher proliferative potential than PGCs, supporting the hypothesis that VSELSCs originate from EpibCs through AD. The process of SC division, which involves telomere shortening, influences their longevity. In the following section, we will explore the characteristics of SC divisions and how they differ from CSCs and LSCs.

## HE and division types of SCs and MSCs

### SCs

Research on the HE in human EpibCs, encompassing the processes of SD and AD, remains active. Initially identified by Hayflick in the context of fibroblasts, the HE represents the maximum number of cell divisions a cell can undergo before entering senescence (Hayflick, 1965; Shay and Wright, 2000). This limit applies to the total number of divisions a cell can perform, irrespective of whether they are SD or AD. The mechanisms governing SD and AD may differ between EpibCs, which differentiate into all body tissues, and other cell types capable of SD and differentiation (Yu et al., 2007; Nichols and Smith, 2012). Research on SCs, particularly HSCs and neural SCs, indicates that ADs are crucial for their long-term SD and for evading the HE. ADs can decelerate telomere shortening within SC populations, thereby delaying the depletion of the SC pool. Unlike SDs, where both daughter cells inherit equally shortened telomeres leading to accelerated senescence, ADs allow one daughter cell to retain longer telomeres. This mechanism enables SCs to sustain their ability to divide over extended periods, effectively circumventing the typical HE by ensuring that at least one daughter cell maintains telomeric integrity and “youthfulness.” The differences in the HE between SDs and ADs primarily arise from the distinct objectives of these division processes (Neumüller and Knoblich, 2009; Mukherjee et al., 2014; Katajisto et al., 2015). Telomere shortening is a fundamental mechanism underlying the HE. During SDs, telomeres progressively shorten with each division, propelling cells closer to cell death. Conversely, ADs preserve one stem daughter cell with maintained telomeric length, thereby delaying senescence. Additionally, environmental factors can influence whether cells undergo SD or AD, affecting their approach to division and senescence or cell death (Scadden, 2006; Li and Clevers, 2010; Liu and Rando, 2011).

Furthermore, the asymmetric segregation of organelles, such as mitochondria, plays a role in cellular aging and may impact the HE. Cells inheriting older mitochondria tend to age more rapidly and are more likely to differentiate, whereas those with newer mitochondria retain greater SD capacity, prolonging their lifespan and delaying senescence. In the ADs of SCs, daughter cells that inherit older mitochondria are more prone to differentiation and aging, potentially accelerating their reaching the HE (Seo et al., 2018).

### MSCs

MSCs, including those derived from EpibCs, can escape the HE, granting them the capacity for limitless division. They achieve this through several mechanisms that enable them to bypass cellular aging. A primary method by which MCs sustain unchecked growth is telomerase reactivation. Telomerase levels are low in normal somatic cells, resulting in telomere shortening after a finite number of divisions, which triggers cellular aging or programmed cell death. However, telomerase is often overexpressed in MCs, continuously replenishing telomeres, thereby preventing aging and facilitating indefinite division. Additionally, mutations in genes encoding proteins that regulate the cell cycle allow MCs to circumvent the controls that typically inhibit unchecked proliferation, leading to rapid and uncontrolled growth (Harley et al., 1990; Kim et al., 1994; Shay and Wright, 2010; Hanahan and Weinberg, 2011).

Not all MCs rely on telomerase to maintain telomere length. Some cancers utilize alternative mechanisms, such as the alternative lengthening of telomeres (ALT) pathway, which enables certain tumor types and immortalized cells to sustain their telomeres without telomerase activation (Bryan et al., 1997). The ALT pathway operates through homologous recombination, using telomere sequences from other chromosomes as templates to extend telomere length (Cesare and Reddel, 2010). Unlike telomerase, which directly adds telomere repeats, ALT involves the exchange of genetic material between telomeres to achieve elongation (Henson et al., 2002). This mechanism is frequently observed in mesenchymal tumors, such as bone sarcomas, and can also be found in gliomas and neuroendocrine cancers (Henson et al., 2002). Cells employing the ALT pathway typically exhibit telomeres of varying lengths—some significantly shorter and others considerably longer than typical telomeres (Sobinoff and Pickett, 2017). ALT^+^ cells also exhibit distinctive molecular features, including ALT-associated PML bodies, which are nuclear structures that mark ALT activity and play an important role in homologous recombination (Henson et al., 2002). While the ALT mechanism is less common than telomerase activation, it represents a significant strategy for tumors that do not utilize telomerase, enabling them to bypass the HE and divide indefinitely (Cesare and Reddel, 2010). Understanding the ALT mechanism is vital for cancer treatment, as targeting the ALT pathway or its key components may offer new therapeutic options for patients with ALT^+^ cancers (Bryan et al., 1997).

SDs in healthy cells contribute to increasing the number of SCs, while excessive SD in MCs can drive uncontrolled cell growth. ADs are essential for maintaining tissue homeostasis and enabling cell differentiation (Morrison and Kimble, 2006; Cesare and Reddel, 2010). However, AD is often disrupted in cancerous cells, promoting tumor development. MCs can utilize both SD and AD mechanisms to regulate their population, but they do so in a dysregulated manner that accelerates malignant progression (Shahriyari and Komarova, 2013; Chao et al., 2024).

In MCs, these divisions facilitate diversity within the tumor and often involve switching between SDs and ADs in response to environmental cues and cellular needs. ADs play a crucial role in generating differentiated MCs, which can either contribute to MC mass or, in some cases, undergo apoptosis (Tassan et al., 2017; Castro-Oropeza et al., 2018; Majumdar and Liu, 2020; Hitomi et al., 2021; Li et al., 2022; Chao et al., 2024). This division type ensures that not all MCs remain stem-like, allowing for a mixture of rapidly dividing and more differentiated cells. If damaged or stressed, differentiated cells may activate apoptotic pathways, thereby modulating cell growth and preventing the overpopulation of undifferentiated cells. Studies have shown that disruptions in the balance between SDs and ADs can lead to malignant progression, as the regulation of differentiation and apoptosis is compromised. This phenomenon has been observed in various malignancies, including gliomas and mammary tumors, where AD helps control MSC populations and contributes to the heterogeneity of the tumor microenvironment (Tassan et al., 2017; Castro-Oropeza et al., 2018; Majumdar and Liu, 2020; Hitomi et al., 2021; Li et al., 2022; Chao et al., 2024). Similar observations have been made in AML (Bonnet and Dick, 1997).

In the context of EpibCs, both SDs, which maintain the SC pool, and ADs, which lead to the formation of PSCs, are subject to the HE. EpibCs have a finite number of divisions, and when they exhaust their division capacity, they typically enter replicative senescence. However, research has suggested that EpibCs may regulate the HE in a niche-dependent manner under certain conditions. HE differences between SD and AD in EpibCs may involve the ability of some progeny cells to replicate longer or undergo faster aging depending on their function within the organism (Santoro et al., 2016; Chan et al., 2022; Fujino et al., 2022). ADs are vital in regulating proliferation and differentiation, as well as maintaining tissue homeostasis. In contrast, SDs maintain the SD ability of SCs, but each SD shortens telomeres, bringing the cell closer to senescence. In AD, one of the daughter cells remains in a stem state, while the other PSC differentiates and can undergo fewer replications than the mother cell. Telomeres, which shorten with each division, play a crucial role in determining this limit in EpibCs. In both SD and AD, telomere shortening ultimately leads to reaching this limit, causing the cell to enter senescence (maintaining metabolic activity without the ability to divide) and/or apoptosis (Santoro et al., 2016; Fujino et al., 2022).

### Molecular switch

Molecular mechanisms that control the switch between SD and AD in SCs are crucial for balancing SC SD with their differentiation into specialized cells (Knoblich, 2010). In AD, one SC divides into another SC and a PSC that will differentiate into a specific cell type. The transition between SD and AD is regulated by several molecular processes, including cell polarization (complexes formed by partitioning defective 3 homolog/partitioning defective 6 homolog alpha/atypical protein kinase C [PAR3/PAR6/aPKC] polarize the SC, leading to an unequal distribution of organelles and proteins during mitosis and ensuring that one daughter cell retains SC characteristics while the other begins differentiating), the SC niche (SCs closer to their niche tend to undergo AD, while those farther away are more likely to divide symmetrically), signaling pathways (pathways such as Notch, Wnt, Hippo, and Hedgehog are involved in regulating the balance between SD and AD [e.g., Notch pathway activation is essential for AD in various SC types]), and metabolic shifts in mitochondrial activity. This balance between SD and AD is essential for organismal development, tissue repair, and aging. Disruptions in this mechanism can contribute to malignancies, as uncontrolled SD may lead to an accumulation of undifferentiated cells, increasing the risk of tumorigenesis (Chhabra and Booth, 2021; Bolkent, 2024).

The HE refers to the concept that human somatic cells can only divide a certain number of times before they cease to divide and enter senescence or undergo apoptosis. This limit is tied to the gradual shortening of telomeres, which degrade with each cell division. In SD, both daughter cells are nearly identical; thus, both inherit telomeres that shorten equally. Consequently, somatic cells undergoing SDs can undergo a limited number of divisions since their telomeres eventually reach a critically short length, triggering cellular aging. In contrast, AD allows SCs to bypass the constraints of the HE (Lupatov and Yarygin, 2022). In AD, one daughter cell remains an SC with the ability to SD, while the other differentiates into a specialized cell, enabling SCs to continue dividing long-term since they often have active telomerase (Zhang and Li, 2008). Additionally, during AD, SCs can eliminate damaged proteins or organelles by transferring them to the differentiating cell, thereby avoiding the accumulation of damage that could accelerate aging. Some studies suggest that SCs can evade the HE through AD, enabling them to divide for extended longevity (Morrison and Kimble, 2006). SCs tend to exhibit higher levels of telomerase activity, preventing the shortening of their telomeres and allowing them to undergo numerous divisions without aging, unlike their progeny, which, under SD, experience telomere shortening and cellular aging. Evidence indicates that SCs can bypass the HE through mechanisms such as telomerase activity and the segregation of cellular damage during division.

## Epithelial-mesenchymal (EMT) and mesenchymal-epithelial (MET) transition: indicators of cellular plasticity

While EMT and MET exemplify cellular plasticity, their occurrence does not inherently signify pluripotency. Pluripotency refers to a cell’s ability to differentiate into various cell types across different germ layers, whereas EMT and MET involve transitions between two distinct cellular phenotypes. During EMT, structured and polarized epithelial cells within tissues transform into mesenchymal cells, which exhibit enhanced mobility and invasive capabilities. This transition is often associated with cancer metastasis (Sacco and Gomez, 2024). Conversely, MET is the reverse process, where mesenchymal cells revert to the more organized epithelial state. EMT and MET are critical for numerous developmental and healing processes in non-malignant human cells. EMT facilitates the loss of epithelial polarity and the gain of motility in epithelial cells, which is essential during early embryogenesis, organogenesis, and wound healing. For example, EMT plays a vital role in the formation of the heart, somite, and the primitive streak during embryonic development. In contrast, MET is involved in generating epithelial structures from mesenchymal cells during development, such as the kidney tubules and the epithelialization of somite. Beyond embryonic development, EMT is crucial for adult tissue repair. During wound healing, epithelial cells at the wound edge undergo EMT to transition into a mesenchymal state, enabling their migration and facilitating wound closure. Once healing is complete, MET restores the epithelial characteristics of these cells. The ovarian surface epithelium also undergoes EMT during post-ovulatory repair in each menstrual cycle (Panchy et al., 2020). Recent studies on non-tumorigenic epithelial cells have demonstrated that EMT and MET can be induced by factors such as TGF-β, highlighting their reversible nature and role in cellular plasticity during development and regeneration (Lai et al., 2020). These findings emphasize that while EMT and MET are extensively studied in cancer, they also play fundamental roles in normal cellular functions in humans. EMT and MET have both been observed in human lung cancer cells, particularly the A549 cell line, underscoring their importance in cancer progression and metastasis. In lung cancer, research has indicated that EMT and MET contribute not only to its advancement but also to its development of resistance to therapies (Ren et al., 2013; Pastushenko and Blanpain, 2019). In the A549, we have identified CSLC characteristics (Sabisz and Skladanowski, 2009), demonstrated its capacity for EMT (Serocki, 2016), and identified a SD population resembling VSCSLCs or SpLCs (Lica and Pradhan, 2023). The presence of the VSCSLC fraction accounts for the cell line’s plasticity and ability to alternate between EMT and MET. Considering that cells capable of transitioning between EMT and MET originate from a common progenitor, it raises the question of whether this progenitor is highly plastic or represents a less differentiated pluripotent stage.

## Model of HL60 stage development

Independent laboratory studies have identified PSCs in adult human tissues that exhibit cytological and immunophenotypic characteristics indicative of multipotency or pluripotency. Examples of these cells include VSELSCs, MIAMICs, MUSECs, CD133^+^ PSCs and SpLCs (Bhartiya et al., 2022). While it is plausible that these cell types represent the same developmental stage, epigenetic differences arising from their origin or culture conditions result in observable variations (Rieske et al., 2005). The existence of these small PSCs may account for cellular plasticity observed in various tissues. Their diminutive size and capacity to fuse with other cells might explain why they have historically been overlooked in research. It has been hypothesized that these small PSCs are generated during embryogenesis in humans and function as a regenerative reserve. This reserve likely decreases with age due to accumulated life-induced damage and physiological changes. Primitive cell fusion, wherein immature progenitor or stem SCs merge to form multinucleated cells, is integral to numerous regenerative and developmental processes. It also plays a role in the formation of immunological multinucleated giant cells (Palovuori et al., 2004; Erenpreisa et al., 2014; Platt and Cascalho, 2019; Dörnen et al., 2020; Vainshelbaum et al., 2022; Vinogradov and Anatskaya, 2023). Cell fusion is critical even during the embryonic stage (Ogle et al., 2005; Ying et al., 2008). For example, the fusion of ELSCs with somatic cells is essential for the reprogramming of somatic cell nuclei (Tada et al., 2001; Cowan et al., 2005), as well as for mediating pluripotency (Yu et al., 2007). Primary bone marrow cells, established during embryonic development, demonstrate high plasticity and can differentiate into various cell types, including blood, bone, and nerve cells. An early population of LT-HSCs exhibits morphological and functional similarities to very small PSCs. Due to ethical constraints, detailed studies on primitive human EpibCs are limited; however, *in vivo* research provides valuable insights. During the 1980s and 1990s, advancements in culturing HSCs *in vitro* were achieved using specific cytokines such as stem cell factor (SCF), interleukin 3 (IL3), and erythropoietin (EPO) to promote their growth and differentiation. In the 1990s and 2000s, efforts to expand healthy HSLC populations under laboratory conditions demonstrated that environmental manipulation could increase HSLC counts, which had significant implications for transplantation and gene therapies (Jones et al., 1990; Krause et al., 2001). In 2012, one study showed an increase in the number of progenitors (Csaszar et al., 2012). Despite the remarkable regenerative potential of the human hematopoietic system, research has suggested that these cells might lose viability over time, unlike MCs, which appear less affected, particularly among SCs. Pathogenic HSCs initiating AML and pathogenicity might originate during embryogenesis (Marcucci et al., 2011; Cazzola et al., 2021; Barone et al., 2023; de Smith and Spector, 2024). Differentiation of pluripotent stem cells can be effectively applied in disease modeling, particularly when studying developmental processes such as heart development (Chi et al., 2024). This approach allows for identifying disease mechanisms, testing potential therapies, and understanding genetic contributions to diseases. A pivotal breakthrough in culturing LSCs *in vitro* involved developing conditions that support their sustained maintenance and differentiation outside the human body. This breakthrough was achieved by investigating various factors and microenvironments that promote LSLC proliferation, enabling long-term maintenance and detailed analysis of their biology and drug responses. One of the most widely used AML cell lines with confirmed LSLCs is HL60, which was derived through leukapheresis from the peripheral blood of a 36-year-old patient with AML (Collins, 1987). Research has demonstrated that a small fraction of PSCs circulates in the bloodstream, suggesting that leukemic compartments may have been present in the sample used to establish the HL60 culture. Recent findings indicate the presence of SD cell stages within this cell line that exhibit cytological features similar to LT-LSCs or VSLSLCs (Lica and Pradhan, 2023).

## Proposed cascade of stage transformation of AML *in vitro*

Research by J. Dick and colleagues on AML development models *in vitro* has elucidated the hierarchical structure of leukemic cells, with LSLCs positioned at the apex. This hierarchical organization is also observed in the HL60 cell line, a widely used model in AML studies. HL60 exhibits both tissue-specific oligopotency—enabling differentiation into cell types such as dendritic cells (Bakri et al., 2005), macrophages, megakaryocytes, and erythrocytes—and a degree of multipotency, allowing differentiation into osteoclast-like cells (Yoneda et al., 1991; Jacobson et al., 2019; Basu et al., 2023). Using HL60, researchers have developed cell density cytological stage profiles, which have proven invaluable as a screening platform for compounds targeting LSLCs and for mechanistic toxicity studies (Lica et al., 2018). These profiles facilitate the identification of therapeutic agents that specifically target the stem-like population within AML, thereby addressing the root of disease persistence and relapse. Experimental data suggest that AML initiation may occur within pathogenic HSCs through the accumulation of critical mutations, leading to the formation of LSLCs. Pathogenic HSCs likely inherit these mutations from earlier PSCs, specifically HPCs, which acquire pathogenic characteristics during embryogenesis. HSC populations are categorized into LT-HSCs and ST-HSCs; similarly, LSLCs exhibit long-term and short-term stages (Dong et al., 2016; Stauber et al., 2021). HPCs can arise from small MsSCs, which may originate by ADs from VSELSCs or MdSCs, or they might form more directly from VSELSCs, as demonstrated in *in vitro* studies (Suszynska et al., 2016; Shaikh et al., 2017; Bhartiya et al., 2022).

As progenitor developmental stages of AML, these populations should also be present *in vitro*, which has been demonstrated by Lica and Pradhan (2023), who identified VSLSLCs and SLSLCs populations within HL60 cultures, distinguished by their size and CD34 expression. These findings suggest that HL60 retains a hierarchical structure similar to that observed *in vivo*, with distinct subpopulations contributing to disease progression and treatment resistance. In 2023, provided evidence that CSCs arise from oncogenically transformed VSELSCs (Bhartiya et al., 2023b). If this hypothesis is valid, the leukemic equivalents *in vitro* would likely be VSLSLCs. However, due to cytological differences, including cell size, it remains unclear whether LT-HSCs represent more differentiated stages derived from VSELSCs, with their leukemic *in vitro* equivalents potentially being SLSLCs.

An open question is whether VSELSCs represent a distinct developmental stage with ICM blastocyst potential or are phenotypic variants of multipotent (potentially pluripotent) tissue progenitors. Their size distribution falls within the lower part of the Gaussian curve for tissue progenitors, suggesting they might be a specialized subset rather than a separate stage. If pathogenic HPCs accumulate sufficient telomerase-related mutations over their lifespan to drive uncontrolled proliferation and subsequent malignancy, it is plausible that they could proliferate and become malignant within the bone marrow. In advanced stages, these cells might circulate in the bloodstream as transformed VSELSCs (Sovalat et al., 2016; Kuruca et al., 2019). The isolation of HL60 cells via leukapheresis from a patient with advanced AML may explain their presence *in vitro* (Basu et al., 2023).

In *in vitro* AML experiments where cells are cultured in medium at low cell density, proliferating HL60 cells have been observed to regenerate through very small and small cell stages. These stages exhibit cytological features resembling LT-LSLCs or malignant VSELSCs, MsPCs or SpLCs. Unlike ABs and EVs, LSLCs do not stain positively for 7-AAD, and their degree of DNA condensation is more indicative of cellular identity rather than apoptosis or vesicle formation. The immunophenotype of VSLSLCs is characteristic of very primitive, early developmental stages, unlike EVs and ABs, which exhibit a heterogeneous or size-dependent representation of markers associated with cellular maturity, aging, or apoptosis (Das et al., 2024).

**FIGURE 3:**
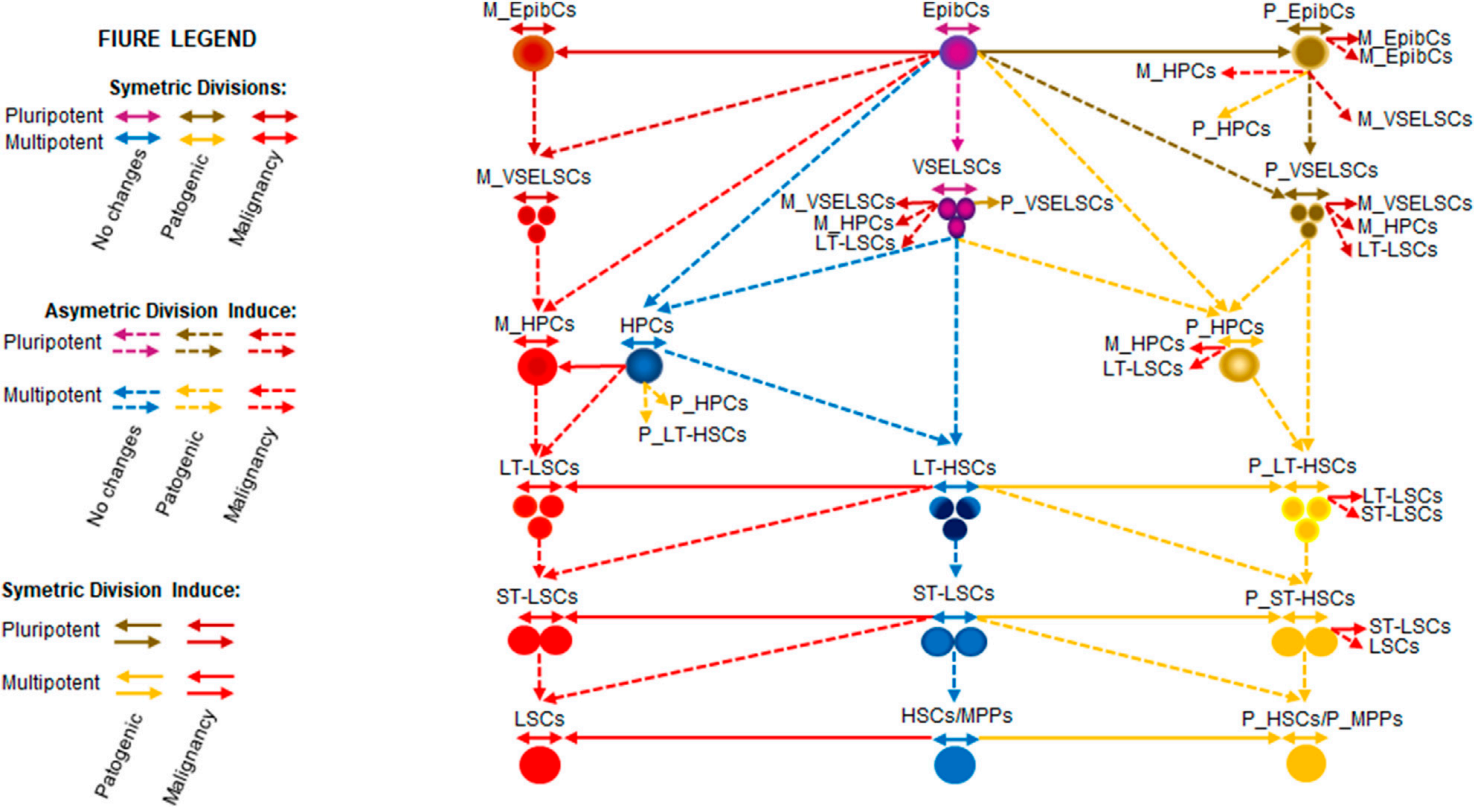
AML initiation and transmission. Disease initiation can occur spontaneously or through pathogenic transmission. The first mutation triggering disease or pathogenicity could have arisen earlier than presented, possibly during the formation of EpibCs from ESCs, or later at the stage of oligopotent progenitors (lacking SD) derived from HSCs/MPPs or P_HSCs/P_MPPs. Disease can independently initiate at various stages. Very small leukemic-like stem cells (VSLSLCs), positioned at the top of the hierarchical development of the HL60 cell line, may originate from M_VSELSCs, M_HPCs, or LT-LSCs. The hypothetical M_HPCs may represent a malignant stage, resembling early progenitors or tissue-specific precursors. The model considers MdSCs arising from VSELSCs or directly from EpibSCs. LT-HSCs, P_LT-HSCs, and LT-LSCs may originate from MdSCs, VSELSCs, or an intermediate hematopoietic precursor not shown in the model. Prefixes denote: M_ for malignancy, P_ for pathogenic. Abbreviations: EpibCs epiblast cells; HSCs, hematopoietic stem cells; HPCs, hematopoietic precursor cells (Anjos-Afonso and Bonnet, 2023); LSCs, leukemic stem cells; MPPs, multipotent progenitor cells; ST-HSCs short-term hematopoietic stem cells; ST-LSCs, short-term leukemic stem cells; LT-HSCs long-term hematopoietic stem cells; LT-LSCs, long-term leukemic stem cells; VSELSCs, very small embryonic-like stem cells.

The model presented in Figure 3, which incorporates VSLSLCs, is based on our framework derived from the hierarchical model of AML for adults proposed by Notta et al. (2011) integrated with the theoretical model suggested by Birnie (1988) along with empirical findings from recent studies (Lica et al., 2018; Lica et al., 2021; Lica and Pradhan, 2023).

Assuming an average cell size of 15 µm for EpibCs during the G1 phase, the deploidization of AD allows 2_3 hypothetical VSELSCs to form in the G1 phase. It is also possible that after undergoing an appropriate number of divisions, EpibCs may differentiate into VSELSCs and deploidize or directly transition into VSELSCs. VSELSCs may also originate from MdPCs. In our stage-based model of HL60 development, we present various models of AML initiation, however we propose that pathogenic EpibCs from the ICM of the early blastocyst might be are responsible for AML origin–pathogenic transmission. Pathogenicity might have also occur earlier in previous developmental stages, such as ESCs, or even originated in gametes—either the sperm or egg—during the parents’ lifetime. Pathogenicity is transmitted (sudden change initiating the disease cannot be excluded either) through pathogenic tissue progenitors, specifically pathogenic LT-HSCs (P_LT-HSCs), pathogenic HPCs (P_HPCs), pathogenic VSELSCs (P_VSELSCs) or their direct precursors, (which might be P_VSELSCs) accumulate mutations and malfunctions over a lifespan. Under SD or AD, they may transform into malignancy M_VSELSCs (establishing VSCSCs or VSLSCs), malignancy HPCs (M_HPCs) or LT-LSCs (*in vitro* compartments of this cellular stages might be VSLSLCs or LT-LSLCs). During leukapheresis from the patient and subsequent adaptation to the *in vitro* environment, the culture flask evolves to support the VSLSLC or LT-LSLC stages on the top of the cellular hierarchy.

Within this model, each primitive stage can trigger the disease and allows SD as well AD, but a critical cell density must be crossed for AD. However, a very intriguing possibility is that VSLSLCs exclusively possess the ability for SD, or their presence is required to facilitate SD in more differentiated SCs. This phenomenon could be associated with cellular fusion, which might also play a key role in a model where VSLSLCs perform only AD, Disease can be initiated independently at any of these stages; however, due to the faster division rates and intensified downstream processes in SCs, initial leukemogenic events are more likely to originate in LSCs/ or lineage-specific progenitors rather than their precursors. Over time, with sufficient lifespan allowing for the accumulation of leukemogenic changes, these events can also occur in LT-HSCs (as well in earlier progenitor stages of lifetime blood development)—initiating AML. LT-LSCs or in LT-HSC precursors, which might be VSELSCs (VSLSLCs). This progression contributes to the aggressive nature of AML relapses (De Haan and Lazare, 2018; Verovskaya et al., 2019; Long et al., 2022). The potential transformation of pathogenic HPCs (potentially pathogenic LT-HSCs, MsSCs or VSELSCs) with accumulated telomerase-related mutations underscores the role of genetic instability in driving uncontrolled proliferation and malignancy within the bone marrow. In advanced stages of AML, these leukemic may circulate in the bloodstream as VSLSCs, which can be isolated via leukapheresis, explaining their presence and behavior *in vitro* (Jia et al., 2019; Cuevas et al., 2024).

## Translational plans for the human condition

One of the primary challenges is obtaining a sufficient number of VSELSCs from adult humans for high-quality RNA sequencing, which arises partly due to ethical constraints and the naturally low abundance of these cells in adult tissues. The scarcity of VSELSCs also complicates efforts to conduct comprehensive molecular profiling, which is essential for understanding their potential and mechanisms of action. Despite these challenges, recent advancements in sequencing technologies offer promising avenues to overcome the limitations associated with VSELSC isolation and analysis. Techniques such as next-generation sequencing, single-molecule sequencing, and innovative methods leveraging feedback effects to amplify SC stages in culture are paving the way for more detailed molecular profiling of VSELSCs. These technological improvements may enable researchers to comprehensively analyze VSELSCs in the near future, thereby enhancing our understanding of their role in cellular plasticity and regenerative processes. The number of VSELSCs in adult tissues decreases with age, suggesting that these cells possess mechanisms to actively shorten their telomeres during SDs. Telomere shortening acts as a biological limit, restricting the potential for feedback amplification methods to expand VSELSCs for regenerative medicine applications (Harley et al., 1990; Cesare and Reddel, 2010; Shay and Wright, 2010; Lupatov and Yarygin, 2022). However, this limitation is less significant when utilizing the feedback effect on VSELSCs for their use in comparative analysis and mechanistic study purposes. This inherent limitation underscores the need to develop alternative strategies to maintain or rejuvenate VSELSC populations without compromising their genomic integrity. VSLSLCs derived from the HL60 cell line represent an MC stage capable of SD and may exhibiting active telomerase activity (Basu et al., 2023; Lica and Pradhan, 2023). Extensively used in AML research, the HL60 generates a high total cellular mass over multiple culture passages. This extensive proliferation indicates a malfunction within the cells, potentially contributing to the malignancies. The active telomerase in VSLSLCs enables these cells to maintain telomere length, thereby bypassing the HE and supporting their unlimited division—a hallmark of cancerous cells (Yamada et al., 2008; Bartoszewska et al., 2024).

Future research should focus on developing methods to efficiently isolate and expand VSELSCs from adult tissues while preserving their genomic stability. Elucidating the precise molecular pathways involved in telomere maintenance and SD in VSLSLCs could also identify potential targets for therapeutic intervention in AML. Collaborative efforts integrating advanced sequencing technologies, SC biology, and oncology are essential to translate these findings into clinical applications that benefit patients with regenerative needs and malignancies. Given the potential for VSLSLCs to undergo unlimited SDs, producing sufficient cells for reliable sequencing is becoming increasingly feasible. Comparative molecular profiling of VSELSCs or VSLSLCs with existing datasets from early human embryonic stages and somatic cells is essential to elucidate their unique and shared characteristics. Data from scRNA-seq of early human embryos (Xue et al., 2013), along with DNA sequences from somatic cells sequenced in the Human Genome Project (HGP), provide robust comparative baselines.

Most HGP data was derived from the peripheral blood lymphocytes of anonymous donors, serving as a representative human reference genome. By comparing these datasets with scRNA-seq results from morula, blastula, gastrula and VSELSC and/or VSLSLC stages, researchers can identify both shared and unique molecular signatures among these cell groups. This comparative analysis is best conducted using set-based approaches alongside differential gene expression analysis techniques (Lachmann et al., 2018). Specifically, examining scRNA-seq data from morula-blastula-gastrula (MBG), HGP lymphocytes, VSELSCs and/or VSLSLCs will allow for the identification of intersections and differences. To systematically identify shared and unique molecular signatures, the following set-based comparisons are proposed. Here, C represents the common elements among the respective datasets:

VSELSCs
• C_MBG_ ∩ VSELSCs = (MBG ∩ VSELSCs) − HGP
• C_VSELSCs_ ∩ HGP = (VSELSCs ∩ HGP) − MBG
• C_MBG ∩ HGP \ VSELSCs_ = (MBG ∩ HGP) − VSELSCs
• C_VSELSCs \ (MBG ∪ HGP)_ = VSELSCs − (MBG ∪ HGP)

VSLSLCs
• C_MBG_ ∩ VSLSLCs = (MBG ∩ VSLSLCs) − HGP
• C_VSLSLCs_ ∩ HGP = (VSLSLCs ∩ HGP) − MBG
• C_MBG ∩ HGP \ VSLSLCs_ = (MBG ∩ HGP) − VSLSLCs
• C_VSLSLCs \ (MBG ∪ HGP)_ = VSLSLCs − (MBG ∪ HGP)

VSELSCs vs. VSLSLCs

• C_VSLSLCs ∩ VSELSCs_ = (VSLSLCs ∩ VSELSCs) − (MBG ∪ HGP)
• C_(VSLSLCs \ VSELSCs)_ ∩ MBG = (VSLSLCs ∩ MBG) − VSELSCs
• C_(VSLSLCs \ VSELSCs)_ ∩ MBG = (VSELSCs ∩ MBG) − VSLSLCs
• C_(VSLSLCs \ VSELSCs)_ ∩ HGP = (VSLSLCs ∩ HGP) − VSELSCs
• C_(VSLSLCs \ VSELSCs)_ ∩ HGP = (VSELSCs ∩ HGP) − VSLSLCs
• C_VSLSLCs \ (VSELSCs ∪ MBG ∪ HGP)_ = VSLSLCs − (VSELSCs ∪ MBG ∪ HGP)
• C_VSELSCs \ (VSLSLCs ∪ MBG ∪ HGP)_ = VSELSCs − (VSLSLCs ∪ MBG ∪ HGP)
• C_(VSLSLCs ∩ MBG) \ VSELSCs_ = (VSLSLCs ∩ MBG) \ VSELSCs
• C_VSELSCs \ (VSLSLCs ∪ MBG ∪ HGP)_ = VSELSCs \ (VSLSLCs ∪ MBG ∪ HGP)

These analyses will elucidate the molecular characteristics that differentiate VSELSCs and VSLSLCs from other cell types, advancing our understanding of their roles in normal physiology and malignancy (Groff et al., 2019). Hierarchical clustering and dimensionality reduction techniques will clarify expression pattern similarities and differences, while functional and gene set enrichment analyses will identify specific biological processes and pathways involved. Correlation and network analyses will further uncover key genes and interactions driving the unique properties of VSELSCs and VSLSLCs.

### Preclinical and clinical applications

VSLSLCs can assist in identifying the most effective potential AML chemotherapeutic agent by testing half-maximal inhibitory concentrations for cell viability (Lica et al., 2018; Lica et al., 2021; Lica and Pradhan, 2023). These findings are relevant in both preclinical and clinical trials—provided that *in vivo* counterparts are included—and aid in selecting the most efficient and least toxic drugs. Moreover, such studies may enhance our understanding of metabolic pathways applicable to therapy.

Identifying specific markers and mechanisms that lead to VSELSCs transforming into MCs could enable targeted therapies to be developed, which could precisely attack MCs during the early stages of their development, potentially being more effective than current treatments. Furthermore, understanding the characteristics of VSELSCs as precursors to CSCs and LSCs could lead to the development of new biomarkers for earlier detection of leukemia and other cancers. New therapeutic opportunities emerge by characterizing VSELSCs as precursors to CSCs and LSCs. This research could result in therapies that more effectively eliminate cancers at their earliest stages and may also help elucidate the mechanisms by which leukemic and cancer cells resist treatment.

### Molecular and epigenetic mechanisms in the transformation of VSELSCs into CSCs

The transformation of VSELSCs into CSCs involves a multifaceted process driven by genetic and epigenetic alterations. VSELSCs, which express hormone receptors and are susceptible to environmental stress, can undergo global hypomethylation and loss of imprinting at specific loci, leading to their transformation into CSCs (Jones and Baylin, 2007; Polyak and Weinberg, 2009; Ratajczak et al., 2009; Easwaran et al., 2014; Bhartiya et al., 2023b). Epigenetic changes, such as dysregulated DNA methyltransferases and the altered expression of stemness markers such as OCT4, SOX2, and NANOG, play a crucial role in this transformation (Bhartiya et al., 2023b). The transition from quiescent VSELSCs into proliferative CSCs involves acquiring a CSC phenotype, genomic instability, and the activation of oncogenic pathways, ultimately contributing to cancer initiation and progression. Targeting these molecular mechanisms offers a promising approach to prevent or reverse CSC development and effectively combat cancer (Jones and Baylin, 2007; Polyak and Weinberg, 2009; Ratajczak et al., 2009; Easwaran et al., 2014; Bhartiya et al., 2023b).

### Extend the knowledge about carcinogenesis: epigenetic analysis of cells

Previous research on MSCs has primarily focused on more developed MC stages. However, studying VSELSCs, which are earlier precursors and potentially the most primitive cell stage throughout the human lifespan can provide new insights into the initial stages of carcinogenesis—processes that remain poorly understood. Investigating the differences between healthy and leukemic or cancerous VSELSCs, such as VSLSLCs and VSCSLCs, can reveal molecular and epigenetic mechanisms responsible for leukemia and cancer transformation. This understanding can aid in identifying new diagnostic markers and therapeutic targets.

Epigenetic analysis of VSELSCs can elucidate the specific epigenetic changes leading to their transformation into MCs, thereby facilitating the identification of the early stages of MC development. For example, DNA methylation patterns and histone modifications unique to transformed VSELSCs could serve as biomarkers for early diagnosis. By comprehensively and/or VSLSLCs understanding the epigenetic mechanisms regulating VSELSC functions, researchers can develop novel epigenetic therapies to reverse or inhibit malignant transformation. Studying interactions between VSELSCs and their microenvironment can reveal how these cells adapt to different conditions, which is crucial for understanding the mechanisms behind leukemia and cancer treatment resistance. These cellular interactions are potential targets for new therapies preventing the adaptation and survival of MCs under therapeutic pressure.

## Conclusion

Drawing an analogy to developmental stage-dependent transformations, consider the study of silkworm (*Bombyx mori*) instars under controlled conditions that shorten their developmental cycle. Even with accelerated development, silkworms still progress through the egg, caterpillar, pupa, and moth stages. However, the duration of each stage is reduced; for example, caterpillars can transition into pupae without undergoing the complete range of instars (Tomital et al., 2003; Goldsmith et al., 2005; Zhou et al., 2012; Ruiz et al., 2018; Jha et al., 2022). If VSELSCs serve as precursors to SCs and CSCs, it is highly likely that analogous precursor cell stages would be present in continuously growing cell cultures despite the accumulation of various changes and alterations. Taking AML as an example, using the HL60 cell line, it can be inferred that VSLSLCs represent a form of LT-LSLCs or their smaller-sized precursor. Given the availability of experimental material in the form of cell cultures, their increased proliferation compared to healthy cells, and the efficient method for enriching cultures with primitive stages, obtaining sufficient cells for single-cell scRNA-seq (transcriptomics) is possible. This analysis typically requires a minimum of 5,000–1,0000 cells to initiate the reaction (Danielski, 2023). The results from such studies could enhance our understanding of the molecular mechanisms underlying malignant proliferation and growth. They can also shed light on differences in cell division behaviors (e.g., the HE) between healthy VSELSCs and transformed VSLSLCs and VSCSLCs.
